# Self-care practices and associated factors in people with Hansen’s disease in Primary Care*

**DOI:** 10.1590/1518-8345.7921.4961

**Published:** 2026-10-07

**Authors:** Michelle Santos Macêdo, Nanielle Silva Barbosa, Olívia Dias de Araújo, Fernanda Lorrany Silva, Lídya Tolstenko Nogueira, Telma Maria Evangelista de Araújo

**Affiliations:** 1 Universidade Federal do Piauí, Departamento de Pós-Graduação em Enfermagem, Teresina, PI, Brazil.; 2 Scholarship holder at the Coordenação de Aperfeiçoamento de Pessoal de Nível Superior (CAPES), Brazil.

**Keywords:** Practice, Self-care, Leprosy, Primary Health Care, Nursing, Early Diagnosis

## Abstract

**Objective:**

to analyze self-care practices and associated factors in people with Hansen’s disease in Primary Health Care.

**Method:**

a cross-sectional study was conducted in Basic Health Units with 133 cases of Hansen’s disease. The instrument “Attitude and Practice of Self-Care in Leprosy - face, hands and feet” was used. The chi-square and/or Fisher’s exact test, with Yates correction, was used to analyze the association between variables. Logistic regression was applied to analyze self-care practices, with Odds Ratio and respective confidence intervals.

**Results:**

males predominated (54.1%), who were 20 to 59 years old (62.4%), married (60.9%), and income of one to two minimum wages (48.9%). The attitude towards self-care was positive (98.5%). Among the participants, only 4.5% performed self-care practices related to the face, hands (12.8%) and feet (5.3%). Having plantar or palmar ulcers and neuritis was associated with a reduced likelihood of unsatisfactory self-care.

**Conclusion:**

the attitude towards self-care was positive in the vast majority of participants, showing dissonance with self-care practices, which were predominantly unsatisfactory. Self-care in Hansen’s disease is essential for the prevention of sequelae and requires that healthcare professionals develop continuous guidance and monitoring, aiming at improving quality of life.

## Highlights

**(1)** The impact of Hansen’s disease and its influence on a person’s self-care.

**(2)** The practice of self-care in maintaining a person’s functional integrity.

**(3)** Disabilities caused by the disease can lead to limitations.

**(4)** The importance of self-care in preventing physical disabilities.

**(5)** Understanding of self-care among people affected by the disease.

## Introduction

Hansen’s disease is one of the oldest diseases known to humankind, originating in West Africa about 100,000 years ago and spreading around the world through people traveling on trade routes and through colonialism. It primarily affects the peripheral nerves and skin, but it can also affect other organs systemically^1^ ^)-(^ ^3^.

The number of new cases worldwide in 2022 was 174,087, corresponding to a detection rate of 21.8 cases per 1 million inhabitants. India, Brazil, and Indonesia reported more than 10,000 new cases of Hansen’s disease each, and in this classification, these countries, including Brazil, remain priorities for the World Health Organization (WHO) due to the number of new cases^2^.

Brazil is a country with a high burden of Hansen’s disease and has not reached the elimination targets set by the WHO, including early diagnosis^3^. From this perspective, the Degree of Physical Disability (DPD) is an epidemiological indicator used in the evaluation of the disease surveillance program and determines the earliness of diagnosis^4^ ^)-(^ ^5^.

The physical disabilities acquired by late diagnosis of Hansen’s disease, mostly DPD 2, affect the physical body, family and social life. Self-care is the main strategy, at the national and global levels, which should be widely reinforced from diagnosis onwards, based on procedures, techniques and exercises that minimize and/or prevent the effects related to the eyes, face, upper and lower limbs, considering the main sites affected by the disease^5^ ^)-(^ ^8^.

The disabilities and deformities caused by Hansen’s disease can cause limitations, interfere with the ability to work, social and family life, as well as result in psychological problems related to stigma and prejudice that can isolate them from social interaction^9^. Therefore, it is important to know the level of understanding of the affected people about self-care, so that actions can be implemented to prevent and/or minimize disabilities.

Hansen’s disease excludes the individual from their community, due to the stigma and prejudices that still exist, in addition to the lack of investment in the area of research and development of new technologies for treatment and cure. Considering this problem, in 2024, the Healthy Brazil Program - Uniting to Care was launched in the country, with the purpose of promoting intersectoral actions that contribute to the elimination and reduction of diseases and infections determined and perpetuated by poverty, hunger and social inequities, among them Hansen’s disease^10^ ^)-(^ ^11^.

Insufficient knowledge of people is the main cause of fear and prejudice, even though the disease is curable. It is believed that adherence to guidelines requires educational actions carried out by professionals within the Family Health Strategy (FHS), especially nurses, and that these should begin at the time of diagnosis, so that the patient is informed and clarified about prevention and self-care activities to be practiced throughout life^1^ ^),(^ ^12^.

This study provides important information on the self-care practices of people with Hansen’s disease, opening the possibility for professionals to instrumentalize the practice in controlling the disease in a more appropriate way, emphasizing guidelines on self-care, which will surely reflect in the better quality of life of the affected people. Thus, the contributions to the health service will be effective in encouraging actions that prevent complications, such as physical disabilities, which reverberates in contributions to quality of life. From this perspective, this study aimed to analyze self-care practices and associated factors in people with Hansen’s disease in Primary Health Care (PHC).

## Methods

### Study design

This is a cross-sectional study, reported in accordance with the recommendations of Strengthening the Reporting of Observational studies in Epidemiology (STROBE)^13^.

### Study setting

The study was conducted in the Basic Health Units (BHU) of the city of Campo Maior-PI, which has a resident population of 45,793 inhabitants and is located in the northern region of the state, 82 km from the capital, Teresina^14^. The city has 18 PHC with 21 FHS teams, 15 in the urban area and six in the rural area. All units participate in the *Previne Brasil* Program, whose main objective is to promote the expansion of access and the improvement of the quality of Primary Health Care. 

### Period

Data collection took place between October and December 2023.

### Population

The population consisted of all individuals affected by Hansen’s disease and under follow-up at the Family Health Strategy (FHS) in the city of Campo Maior (n=145), whose cases were reported to the Notifiable Diseases Information System (SINAN) between January 2019 and December 2023. The choice of a five-year interval considered the time frame recommended by the Ministry of Health (MH) for contact tracing^1^. Out of the total cases, three could not be located, six died from causes unrelated to Hansen’s disease, and three showed signs of dementia due to advanced age, resulting in a final study participant count of 133. 

### Inclusion/exclusion criteria

Individuals undergoing Hansen’s disease treatment or in the post-treatment evaluation phase were included, regardless of age and sex. Individuals with signs of dementia due to advanced age were excluded.

### Study variables

The dependent variable was self-care practice, with binary outcomes: unsatisfactory and satisfactory. The independent variables were all others, which were classified as sociodemographic (sex, race/color, age, marital status, education, occupation, average monthly income, access to social programs, current housing situation, family members residing in the same house, and religion); clinical/epidemiological (time since diagnosis, dose of single multidrug therapy (MDT), operational classification, bacilloscopy, clinical form, degree of physical disability at diagnosis, Hansen’s disease reactions, onset of the reactional episode, plantar/palmar ulcers, associated diseases, and neuritis); guidance received; attitudes towards self-care in Hansen’s disease (whether observing and caring for the face, hands, and feet is necessary); and limiting factors of self-care. 

### Study instrument

The instrument Attitude and Practice of Self-Care in Hansen’s Disease: Face, Hands and Feet (APAHansen, acronym in Portuguese) was used. The items of the instrument were submitted to content validation by a committee composed of eight judges. The overall CVI of the instrument was 0.91 for clarity and 0.96 for relevance of the items, evidencing satisfactory content validity. The intensity of agreement between the judges was measured using the Kappa Index (K), obtaining a value ≥ 0.90, which indicates excellent or almost perfect agreement^15^.

The APAHansen is composed of two parts. The first contains data on the sociodemographic profile and the second addresses the guidance received, attitudes, practices and limitations for self-care. Regarding self-care attitudes, there are six questions and in each of the alternatives, whose connotations are positive, a numerical value ranging from zero to two was assigned, with zero corresponding to the alternative “No”; One point for “Does not wish to answer” and two points for “Yes”, with the most positive attitude totalling 12 points, which corresponds to 100%. Thus, the attitude was classified as follows: 1 to 74% - Negative attitude; 75 to 100% - Positive attitude^15^.

The section on self-care practices contains 46 questions divided into three dimensions: 13 questions for the face, 11 for the hands, and 22 for the feet. For each dimension, the arithmetic mean of the scores was calculated, allowing the initial classification of the participants’ self-care practices into three categories: unsatisfactory (average of 1.0 to 2.5 points), needs improvement (average of 2.6 to 3.5 points), and satisfactory (average of 3.6 to 5.0 points). Subsequently, these variables were recategorized in order to facilitate statistical analyses, considering that the “needs improvement” category does not yet represent a satisfactory level of self-care. Thus, self-care practices were dichotomized according to the following cut-off points: unsatisfactory (average of 1.0 to 3.5 points) and satisfactory (average of 3.6 to 5.0 points)^15^.

### Data collection

Data collection was carried out in person, from October to December 2023, through the application of the form individually and in a location that guaranteed comfort, confidentiality, and privacy to the participant, after prior contact, which occurred by telephone or in person, during appointments at the Primary Health Care Units (PHC).

After authorization from the Municipal Health Department to use the SINAN data and approach the study participants at the PHC, the researcher made initial contact with the nurses at the PHC. In this first moment, strategies for operationalizing data collection were discussed (dates, times, and access to SINAN).

Data on the clinical and epidemiological profile of the participants were obtained directly from the SINAN database, referring to the time of diagnosis, dose of treatment with MDT-U, operational classification, bacilloscopy, clinical form, degree of physical disability at diagnosis, Hansen’s disease reactions, onset of the reactional episode, palmar/plantar ulcers, associated diseases, and neuritis.

Participants’ consent to participate in the study was obtained through an Informed Consent Form (ICF) and an Informed Assent Form (IAF). 

### Data processing and analysis

The collected data were exported to the Statistical Package for the Social Sciences (SPSS) version 26. Descriptive analysis was used, with absolute and percentage frequency distributions, measures of position and dispersion. In the association between categorical variables and self-care practices in Hansen’s disease, the chi-square and/or Fisher’s exact test, with Yates correction, was used.

In the study of self-care practices as a response variable, logistic regression was used, with odds ratio (OR) and respective confidence intervals, seeking to explain factors related to the unsatisfactory self-care practice of the study population. The criterion for inclusion of variables in the multiple logistic model was the association at the 20% level (p˂0.200) in the bivariate analysis and in the final model, in turn, the statistical significance was set at 5% (p˂0.05)^16^.

### Ethical aspects

This research was initiated after authorization from the Municipal Health Department of the city under study and approval by the Research Ethics Committee (CEP) of the Federal University of Piauí (UFPI), under opinion number 6.323.688, approved on September 26, 2023.

## Results

Among the 133 participants, 54.1% were male and 75.2% identified as mixed-race. The predominant age range was 20 to 59 years (62.4%), marital status married (60.9%), education level compatible with incomplete primary education (51.1%), occupation retired (28.6%), income between one and two minimum wages (48.9%), no access to social programs (66.2%), homeownership (78.9%), and Catholic religion (79.7%), according to Table 1.

**Table 1:** Sociodemographic profile of people affected by Hansen’s disease and under follow-up in the Family Health Strategy (n* = 133). Campo Maior, PI, Brazil, 2023

Variables	n* (%)	CI-95%^†^	Mean	SD^‡^
Sex				
Male	72 (54.1)	(45.7-62.4)		
Female	61 (45.9)	(37.6-54.3)		
Race/color				
White	23 (17.3)	(11.6-24.4)		
Brown	100 (75.2)	(66.6-81.3)		
Black	10 (7.5)	(3.9-12.9)		
Age range			49.2	17.8
≤ 19 years old	10 (7.5)	(3.9-12.9)		
20-59 years old	83 (62.4)	(54.0-70.3)		
≥60 years old	40 (30.1)	(22.8-38.2)		
Marital status				
Married	81 (60.9)	(30.4-58.8)		
Single/Divorced/Separated/Widower	54 (39.1)	(21.4-50.3)		
Education				
Without formal education	13 (9.8)	(5.6-15.7)		
Incomplete primary education	68 (51.1)	(42.7-59.5)		
Complete primary education	06 (4.5)	(1.9-9.1)		
Incomplete secondary education	12 (9.0)	(5.0-14.8)		
Complete secondary education	27 (20.3)	(14.1-27.7)		
Incomplete higher education	01 (0.8)	(0.1-3.5)		
Complete higher education	06 (4.5)	(1.9-9.1)		
Occupation				
Student	11 (8.3)	(4.5-13.9)		
Housewife	24 (18.0)	(12.2-25.2)		
Retired	38 (28.6)	(21.4-36.6)		
Unemployed	08 (6.0)	(2.9-11.0)		
Other	52 (39.1)	(31.1-47.6)		
Average monthly income				
<1 MW^§^	40 (30.1)	(22.8-38.2)		
1-2 MW^§^	65 (48.9)	(40.5-57.3)		
≥2 MW^§^	11 (8.3)	(3.4-12.0)		
Without income	17 (12.8)	(7.9-19.2)		
Access to social programs				
Yes	45 (33.8)	(26.2-42.2)		
No	88 (66.2)	(57.8-73.8)		
Housing situation				
Own home	105 (78.9)	(71.4-85.2)		
Rented home	15 (11.3)	(6.7-17.5)		
Home provided by someone else	11 (8.3)	(4.5-13.9)		
Other	02 (1.5)	(0.3-4.7)		
People living in the house			2.7	1.2
Religion				
Catholic	106 (79.7)	(72.3-85.9)		
Evangelical	16 (12.0)	(7.3-18.4)		
Spiritist	03 (2.3)	(0.6-5.9)		
No religion	05 (3.8)	(1.4-8.0)		
Other	03 (2.3)	(0.6-5.9)		

*n = Size of the sample; ^†^CI-95% = Confidence Interval for Proportion; ^‡^SD = Standard deviation; ^§^MW = Current minimum wage = R$ 1,320.00, Brazil, 2023 (which would roughly correspond to US$ 255.00 dollars nowadays)

Most (98.5%) stated that they received guidance from professionals on self-care practices at the primary health care unit, with nurses being the professionals cited by 99.2% of respondents.

The attitude of the surveyed population towards self-care was classified as positive in 98.5% of cases and negative in 1.5%. In the classification of self-care practices, three segments were investigated: face, hands, and feet, and in none of them was a predominance of satisfactory practices observed (Table 2).

**Table 2:** Classification of self-care practices of the study sample related to the face, hands, and feet (n* = 133). Campo Maior, PI, Brazil, 2023

Variables	n* (%)	CI-95%^†^	Mean	SD^‡^
Self-care practice - face			2.6	0.59
Unsatisfactory	66 (49.6)	(41.2-58.1)		
Needs improvement	61 (45.9)	(37.6-54.3)		
Satisfactory	06 (4.5)	(1.9-9.1)		
Self-care practice - hands			2.7	0.67
Unsatisfactory	57 (42.9)	(34.7-51.3)		
Needs improvement	59 (44.4)	(36.1-52.8)		
Satisfactory	17 (12.8)	(7.9-19.2)		
Self-care practice - feet			2.8	0.48
Unsatisfactory	39 (29.3)	(22.1-37.4)		
Needs improvement	87 (65.4)	(57..-73.1)		
Satisfactory	07 (5.3)	(2.4-10.1)		

*n = Sample size; ^†^CI-95% = Confidence Interval for Proportion; ^‡^SD = Standard deviation

Among the cases investigated, 95.5% presented the multibacillary operational classification, with negative bacilloscopy for 70.7%, dimorphic clinical form (90.2%), and DPD 1 at the time of diagnosis in 62.4%. The majority never presented a Hansen’s disease reaction (80.5%), and among those who did, 18% were type 1, but without neuritis (88%), and with the onset of the reactional episode observed at the time of diagnosis. Plantar and palmar ulcers were absent in 88% of those investigated. Associated diseases were Diabetes Mellitus (DM) (15.8%) and Systemic Arterial Hypertension (SAH) (30.8%). Other diseases included hyperthyroidism, anxiety, dyslipidemia, and heart disease (Table 3).

**Table 3:** Clinical characterization of Hansen’s disease cases in the study (n* = 133). Campo Maior, PI, Brazil, 2023

Clinical data	n*(%)	CI-95%^†^
Operational Classification		
Paucibacillary	06 (4.5)	(1.9-9.1)
Multibacillary	127 (95.5)	(90.9-98.1)
Bacilloscopy		
Positive	12 (9.0)	(5.0-14.8)
Negative	94 (70.7)	(62.6-77.9)
Not performed	27 (20,3)	(14,1-27.7)
Clinical Form		
Indeterminate	05 (3.8)	(1.4-8.0)
Tuberculoid	02 (1.5)	(0.3-4.7)
Dimorphic	120 (90.2)	(84.3-94.4)
Virchowian	06 (4.5)	(1.9-9.1)
DPD^‡^ at diagnosis		
DPD^‡^ 0	39 (29.3)	(22.1-37.4)
DPD^‡^ 1	83 (62.4)	(54.0-70.3)
DPD^‡^ 2	11 (8.3)	(4.5-13.9)
Hansen’s disease Reaction		
Type I	24 (18.0)	(12.2-25.2)
Type II	01 (0.8)	(0.1-3.5)
Never had	107 (80.5)	(73.1-86.5)
Had at another time	01 (0.8)	(0.1-3.5)
Onset of reactional episode		
Diagnosis	13 (9.8)	(5.6-15.7)
Treatment	08 (6.0)	(2.9-11.0)
Post-discharge	05 (3.8)	(1.4-8.0)
Not applicable	107 (80.5)	(73.1-86.5)
Plantar/Palmar Ulcers		
Current	07 (5.3)	(2.4-10.1)
Previous	01 (0.8)	(0.1-3.5)
Never had	117 (88.0)	(81.6-92.7)
No record	08 (6.0)	(2.9-11.0)
Associated Diseases		
Diabetes Mellitus		
No	112 (84.2)	(77.3-89.6)
Yes	21 (15.8)	(10.4-22.7)
Hypertension		
No	92 (69.2)	(61.0-76.5)
Yes	41 (30.8)	(23.5-39.0)
Other		
No	46 (34.6)	(26.9-42.9)
Yes	87 (65.4)	(57.1-73.1)
Neuritis		
Current	13 (9.8)	(5.6-15.7)
Previous	02 (1.5)	(0.3-4.7)
Not applicable	117 (88.0)	(81.6-92.7)
No record	01 (0.8)	(0.1-3.5)

*n = Sample size; ^†^CI-95% = Confidence Interval for Proportion; ^‡^DPD = Degree of Physical Disability

In the multiple regression model, only two variables remained statistically associated with unsatisfactory self-care practices: having plantar or palmar ulcers was associated with a 10% reduction in the odds of exhibiting unsatisfactory self-care (ORa = 0.90; p = 0.05), and the presence of neuritis reduced the odds by 59% (ORa = 0.41; p = 0.04) (Table 4).

**Table 4:** Logistic models of unsatisfactory self-care practices among people with Hansen’s disease (n* = 133). Campo Maior, PI, Brazil, 2023

Variables	Bivariate analysis				Multiple analysis			
	GOR*	CI 95%^†^		p- value^||^	AOR^¶^	CI 95%^†^		p- value^||^
		LL^‡^	UL^§^			LL^‡^	UL^§^	
Sociodemographic data								
Sex				0,23				
Male	1.57	0.74	3.33					
Female	1							
Race/Color								
White	1.52	0.32	7.14	0.59				
Brown	1.71	0.45	6.53	0.43				
Black	1							
Marital Status								
Married/Common-law Marriage	0.92	0.44	2.07	0.92				
Divorced/Single/Widowed	1							
Education								
No schooling	1.66	0.19	14.05	0.63				
Elementary School	1.04	0.17	6.09	0.96				
High School	1.50	0.23	9.46	0.66				
Higher Education	1							
Occupation								
Student	0.98	0.22	4.23	0.98				
Housewife	0.89	0.30	2.61	0.89				
Retired	0.70	0.28	1.75	0.70				
Unemployed	1.10	0.19	6.13	1.10				
Other	1							
Average Monthly Income								
<1 Minimum Wage**	0.50	0.12	2.06	0.33				
1-2 Minimum Wages**	0.44	0.11	1.73	0.24				
No income	1							
Operational Classification								
Paucibacillary	-			0.99				
Multibacillary	1							
Clinical Form								
Indeterminate	2.00	0.12	31.97	0.62				
Tuberculoid	-			0.99				
Dimorphous	1.16	0.20	6.65	0.86				
Virchowian	1							
DPD^††^ at Diagnosis								
No	1							
Yes	1.01	0.48	2.48	0.81				
Hansen’s disease Reaction								
No	1				1			
Yes	0.36	0.14	0.88	0.02	-			0.99
Onset of Reactional Episode								
Diagnosis	0.27	0.08	0.89	0.03	-			1.00
Treatment	0.32	0.07	1.375	0.12	-			1.00
Post-Discharge	0.48	0.07	3.04	0.43	-			1.00
Not applicable	1				1			
Plantar/Palmar Ulcers								
No					1			1
Yes	0.38	0.09	1.64	0.09	0.90	0.16	4.80	0.05
Diabetes								
No	1							
Yes	1.04	0.37	2.92	0.93				
Hypertension Arterial								
No	1							
Yes	0.84	0.38	1.88	0.68				
Other								
No	1							
Yes	1.27	0.58	2.75	0.54				
Neuritis								
No	1				1			
Yes	0.31	0.10	0.93	0.03	0.41	0.12	1.37	0.04
Self-Care Attitude								
Negative Attitude	1.51	0.31	1.11	0.99				
Positive Attitude								

*GOR = Gross odds ratio; ^†^CI-95% = Confidence Interval for Proportion; ^‡^LL = Lower limit; ^§^UL = Upper limit; ^||^p-value; ^¶^AOR = Adjusted odds ratio; **MW = Current minimum wage = R$ 1,320.00, Brazil, 2023; ^††^DPD = Degree of Physical Disability

## Discussion

In the studied population, males accounted for the majority of new cases of multibacillary Hansen’s disease, especially in the 20-59 age group, with incomplete primary education and low family income. Hansen’s disease affects susceptible individuals and in endemic countries, mainly those in Latin America, where factors such as low socioeconomic status, malnutrition, dispersed rural population, and limited and inadequate access to health services for diagnosis and treatment, among other characteristics, play an important role in the epidemiological chain of transmission and increase the hidden prevalence of the disease. In addition, economic status can be a possible obstacle to adherence to and completion of treatment^17^ ^)-(^ ^22^.

School is also a determining factor for the effectiveness of health education actions, as it acts as a mediator in the process of understanding the disease and in adherence to treatment. It is understood that individuals with a higher level of education tend to have greater ease in mastering concepts related to prevention and early identification of signs and symptoms, which consequently favors self-care practices and reduces the risk of physical disabilities. A randomized study with 635 people affected by Hansen’s disease in Nigeria concluded that schooling, although it may be related to a better understanding of self-image and coping, may not protect individuals from the stigma experienced in the community. This finding reinforces the need for improved educational programs and campaigns to address misconceptions and improve awareness about Hansen’s disease^23^.

One finding of this study that caught our attention refers to the proportion of multibacillary cases with grade 1 physical disability, which reinforces the hidden prevalence of the disease associated with late diagnosis and/or insufficient preparation of primary health care professionals for the correct and timely diagnosis of cases^24^. A population-based survey conducted in China showed that, despite progress in reducing the prevalence of physical disabilities related to Hansen’s disease between 1990 and 2020, the proportion of cases with disabilities among those affected by the disease remains high, indicating detection at more advanced stages and persistent community transmission^25^. It is essential to qualify and expand early detection, prevention and timely treatment actions, considering that it is the clinical form with the greatest epidemiological importance, as it eliminates a greater number of bacilli, which contributes to maintaining the transmission chain^17^.

Smear microscopy was performed in most cases, obtaining a negative result. This is a complementary laboratory test to the clinical diagnosis, whose objective is to detect the presence of *M. leprae* in intradermal scraping smears and estimate the bacillary load presented by the patient. When positive, it indicates a multibacillary case; when negative, it does not allow ruling out the diagnosis of the disease and its performance is not mandatory for clinical diagnosis. Furthermore, the test helps monitor treatment, making it possible to compare the bacillary index at the beginning and end of treatment^1^ ^),(^ ^26^.

It is noteworthy that Hansen’s disease reactions were absent in most participants, however, in those that did present them, they were type I. The appearance of reactions is one of the main difficulties in the clinical management of people with Hansen’s disease, as they are acute and inflammatory episodes that can occur before, during and after multidrug therapy, and cause irreversible physical disabilities in the nerves when not recognized and treated properly^27^.

The attitude of people affected by Hansen’s disease towards self-care was overwhelmingly positive. On the other hand, unsatisfactory self-care practices predominated. In general, a favorable attitude is expected to be a precursor to a healthy practice. However, there is not always consistency between what people believe and their practical actions, which can be understood as a dissonance between attitude and behavior.

According to the theory of Cognitive Consistency, an attitude is likely a precursor to a pattern of behavior. However, since 1934, La Pierre had already referred to inconsistencies between attitudes and practices, pointing out disparities between what is said and what is done. Reinforcing this idea, it is stated that cognitive incoherence is always present for most people, most of the time, and for all people some of the time. Thus, this approach has been challenged from an empirical and theoretical point of view^28^ ^)-(^ ^29^.

On the other hand, the Contingent Consistency approach analyzes the consistency between the subject’s attitude and practice in relation to the object and the situational circumstances that surround it, pointing out the importance of the support network or structural supports, considering two types of individuals: those who maintain a permanent consistency between attitudes and practices, regardless of the supports, and the ambivalent ones, who change their behavior depending on momentary supports or pressures^29^ ^)-(^ ^30^.

In short, according to this theory of contingent consistency, in order to avoid dissonances between attitudes and practices, it is necessary to consider the situation in which the object is found and, only by controlling negative circumstantial factors, can the subject achieve a positive attitude that provides them with better levels of health.

From this perspective, the execution of self-care is fundamental as a primary factor in the prevention of disabilities and in the person’s responsibility for maintaining their health^9^ ^),(^ ^31^, however, there are contexts of social vulnerability, where structural barriers and lack of resources hinder the materialization of positive attitudes in consistent self-care practices, which may be influenced by skills, limitations, cultural, social and economic conditions that directly interfere with the health of those who perform it^32^. In the study carried out in Jiangsu province, China, the self-care capacity of the groups treated in health services and at home was influenced by self-care skills and health literacy^33^.

Thus, in health, it is not enough to know only the attitudes and practices of the individual in relation to a given situation, but also the contingencies in which he or she is embedded. Therefore, although the affected person may be in favor of self-care, he or she may not understand exactly how to do it or what the basis of its implementation is. In addition, the absence of continuous support and the lack of technical knowledge about the necessary care reinforce this dissociation between attitude and practice, highlighting gaps in health education^34^.

Considering that stigma can be a barrier to self-care, the literature shows that peer support and psychosocial support, through self-help groups, contribute significantly to its reduction among people affected by Hansen’s disease and the community in which they are embedded. These groups can play a key role in interventions to reduce the stigma surrounding neglected tropical diseases, contributing to faster diagnosis, greater adherence to therapy and self-care practices^23^ ^),(^ ^35^.

Hansen’s disease remains a major challenge for national and international public health, considering that it is a disease that affects not only the skin but also the peripheral nerves, with a strong tendency to generate deformities and disabilities, as well as inflammatory processes (neuritis)^36^ ^)-(^ ^39^. It is noteworthy that unsatisfactory practices were reduced when participants presented plantar or palmar ulcers, or neuritis. Self-care demands are directly related to the eyes, face, upper and lower limbs, especially hands and feet, considering that these are the main sites of cutaneous and neural involvement^32^.

Peripheral neuropathy is one of the main manifestations of the disease, as it compromises sensory, motor, and autonomic functions, resulting in visible deformities and impairment of neural function^1^ ^),(^ ^40^. Among the most common physical deformities, plantar ulcers are one of the main ones. The plantar region is the most affected area by ulcers, because people do not perceive physical traumas such as injuries, burns, and the wounds are continuously exposed to the impact of locomotion and contact, due to decreased or loss of sensitivity. Delay in diagnosis is the main reason for the risk of developing disabilities, such as ulcers^41^ ^)-(^ ^42^.

The nurse, as one of the professionals who are part of the primary health care team, becomes a key element in the execution of the actions proposed by the National Program for the Control and Elimination of Hansen’s disease (PNCEH), acting in the comprehensive care of patients, from diagnosis to the completion of treatment, in addition to guiding self-care practices^43^ ^)-(^ ^44^. It is noteworthy that the involvement of trained professionals with effective communication facilitates the early recognition of signs and symptoms, access to health services, encouragement of treatment, and motivation for self-care practices in order to prevent disabilities related to Hansen’s disease^45^ ^)-(^ ^46^.

Contributing to the previous premise, the investigation carried out in the districts of Dhanusa and Mahottari, Nepal, to evaluate the knowledge and skills of 31 local health professionals, concluded that the overall median performance of Hansen’s disease care was low (53%) and could be improved through evidence-based training, face-to-face follow-up, monitoring and supervision, with the aim of facilitating the elimination of Hansen’s disease transmission. However, the study observed that counseling provided by specialized health professionals significantly improved patients’ positive beliefs and practices regarding self-care during the study^47^.

Hansen’s disease, without early intervention, can cause irreversible nerve damage, impairment, and disability, implying not only a health problem but also a social problem. Self-care is highlighted as one of the main strategies in the prevention, monitoring, management, and control of disabilities, with the individual being responsible for maintaining and promoting their own life, health, and well-being^44^ ^),(^ ^48^.

The disabling potential caused by the disease is quite significant, and the assessment of disabilities is important for education and the promotion of self-care. Prevention is carried out through procedures and exercises that the person themselves, properly guided and trained, should perform regularly at home, during treatment, and after discharge. A change in behavior is fundamental, as well as the inclusion of self-care in the routine of affected people^49^.

The use of Educational Technologies contributes to a significant improvement in adherence to self-care of the face, hands, and feet. Furthermore, it improves independence rates during the performance of activities of daily living, increases the frequency of physical and mental self-care practices, and adherence to Hansen’s disease treatment^50^.

One of the limitations identified concerns the approach to people who had Hansen’s disease between 2019 and 2023, that is, within a period of up to five years. During this period, it is natural that changes of address and deaths occurred. Time was also a factor that may have contributed to possible memory biases. In addition, the scarcity of studies on self-care practices in Hansen’s disease implied some difficulties in comparisons; however, it did not harm the study.

This study makes valuable contributions to the field of self-care in Hansen’s disease, especially by reinforcing the need for interventions that go beyond verbal counseling, focusing on demonstrative workshops and longitudinal monitoring of self-care skills that are being developed. It also contributes by highlighting that the focus of programmatic actions should be redirected to patients without physical disabilities or apparent skin lesions, with the aim of breaking the cycle of worsening that can lead to irreversible damage, promoting empowerment in preventing these disabilities through self-care, and enabling access to the necessary resources to carry it out satisfactorily. 

## Conclusion

The study results reveal that most participants affected by Hansen’s disease are embedded in a sociodemographic and economic context of social inequalities, in which low income and low levels of education prevailed. Although no statistically significant association was observed between sociodemographic data and self-care practices for the face, hands, and feet, there may be clinical importance. It is assumed that the adoption and development of these activities are influenced by other factors, such as those related to the health team and subjective variables.

The attitude towards self-care was positive in the vast majority of participants; however, unsatisfactory self-care practices predominated, which were significantly reduced in the presence of plantar or palmar ulcers, or even neuritis. Health education initiatives are key strategies in addressing Hansen’s disease, and should be implemented by professionals in primary health care teams, who play an important role in the comprehensive care of users, both in early identification, assessment and prevention of complications, adherence to treatment and promotion of recovery, as well as in monitoring self-care needs. To this end, it is necessary that these professionals are adequately qualified.

The creation and implementation of mutual support groups is a strategy that provides people affected by Hansen’s disease with a space to share experiences and knowledge, develop their skills, and expand their knowledge about the necessary care, focusing on the prevention of ulcerations, sequelae, and disabilities. 

## Data Availability

All data generated or analysed during this study are included in this published article.
