# Supplementary material for: Self-care practices and associated factors in people with Hansen’s disease in Primary Care
Source: Rev Lat Am Enfermagem. 2026 Oct 7;34:e4961. doi: 10.1590/1518-8345.7921.4961 (PMC13679909; doi:10.1590/1518-8345.7921.4961)
Supplement: Translation

# Translation: Prática de autocuidado e fatores associados em pessoas com hanseníase na Atenção Primária*

**DOI:** 10.1590/1518-8345.7921.4962

**Authors:** Michelle Santos Macêdo, Nanielle Silva Barbosa, Olívia Dias de Araújo, Fernanda Lorrany Silva, Lídya Tolstenko Nogueira, Telma Maria Evangelista de Araújo

**Affiliations:** 1 Universidade Federal do Piauí, Departamento de Pós-Graduação em Enfermagem, Teresina, PI, Brasil.; 2 Bolsista da Coordenação de Aperfeiçoamento de Pessoal de Nível Superior (CAPES), Brasil.

**Keywords:** Prática, Autocuidado, Hanseníase, Atenção Primária à Saúde, Enfermagem, Diagnóstico Precoce

## Abstract

**Objetivo:**

analisar práticas de autocuidado e fatores associados em pessoas com hanseníase na Atenção Primária à Saúde.

**Método:**

estudo transversal realizado em Unidades Básicas de Saúde com 133 casos de hanseníase. Utilizou-se o instrumento Atitude e Prática de Autocuidado na Hanseníase - face, mãos e pés. Na associação entre as variáveis utilizou-se o teste qui-quadrado e/ou exato de Fisher, com correção de Yates. Para análise das práticas de autocuidado, aplicou-se regressão logística, com *Odds Ratio* e respectivos intervalos de confiança.

**Resultados:**

predominou sexo masculino (54,1%), faixa etária 20 a 59 anos (62,4%), casados (60,9%), renda de um a dois salários-mínimos (48,9%). A atitude frente ao autocuidado foi positiva (98,5%). Dentre os participantes, apenas 4,5% realizavam práticas de autocuidado relacionadas à face, mãos (12,8%) e pés (5,3%). Ter úlceras plantares ou palmares e neurites foi associado à redução das chances de autocuidado insatisfatório.

**Conclusão:**

a atitude frente ao autocuidado foi positiva na expressiva maioria dos participantes, mostrando dissonância com as práticas de autocuidado, que foram predominantemente insatisfatórias. O autocuidado em hanseníase é essencial à prevenção de sequelas e requer que profissionais de saúde desenvolvam orientações e monitoramento contínuos, visando à melhoria da qualidade de vida.

## Destaques

**(1)** O impacto da hanseníase e sua influência no autocuidado da pessoa.

**(2)** A prática de autocuidado na manutenção da integridade funcional da pessoa.

**(3)** Incapacidades produzidas pela doença podem levar a limitações.

**(4)** Importância do autocuidado na prevenção de incapacidades físicas.

**(5)** Compreensão das pessoas afetadas pela doença sobre autocuidado.

## Introdução

A hanseníase é uma das enfermidades mais antigas da humanidade, com origem na África Ocidental há cerca de 100.000 anos, espalhando-se pelo mundo por meio das pessoas que se deslocavam em rotas comerciais e pelo colonialismo. Afeta primariamente os nervos periféricos e a pele, podendo também acometer outros órgãos de forma sistêmica^1^ ^)-(^ ^3^.

O número de casos novos em âmbito mundial em 2022 foi 174.087, correspondendo a uma taxa de detecção de 21,8 casos por 1 milhão de habitantes. Índia, Brasil e Indonésia reportaram mais de 10 mil casos novos de hanseníase cada e nessa classificação, esses países, incluindo o Brasil, permanecem como prioritários para a Organização Mundial da Saúde (OMS) pelo número de casos novos^2^.

O Brasil é um país com alta carga para a hanseníase e não atingiu as metas de eliminação determinadas pela OMS, dentre elas o diagnóstico precoce^3^. Nesta perspectiva, o Grau de Incapacidade Física (GIF) é um indicador epidemiológico utilizado na avaliação do programa de vigilância da enfermidade e determina a precocidade do diagnóstico^4^ ^)-(^ ^5^.

As deficiências físicas adquiridas pelo diagnóstico tardio da hanseníase, na maioria GIF 2, afetam o corpo físico, o convívio familiar e o social. O autocuidado é a principal estratégia, nos âmbitos nacional e mundial, que deve ser amplamente reforçado desde o diagnóstico, baseado em procedimentos, técnicas e exercícios que minimizam e/ou previnem os efeitos relacionados aos olhos, face, membros superiores e inferiores, considerando-se os principais sítios acometidos pela enfermidade^5^ ^)-(^ ^8^.

As incapacidades e deformidades ocasionadas pela hanseníase podem causar limitações, interferir na capacidade de trabalho, na vida social e familiar, bem como resultar em problemas psicológicos relacionados ao estigma e preconceito que podem isolá-los do convívio social^9^. Por isso, a importância de se conhecer o nível de compreensão das pessoas afetadas sobre o autocuidado, para que ações sejam implementadas no sentido de prevenir e/ou minimizar as incapacidades.

A hanseníase exclui o indivíduo da sua coletividade, pelo estigma e pelos preconceitos ainda existentes, além da ausência de investimento na área de investigação e desenvolvimento de novas tecnologias para o tratamento e cura. Considerando esta problemática, em 2024, foi lançado no país o Programa Brasil Saudável - Unir para Cuidar, com a finalidade de promover ações intersetoriais que contribuam para a eliminação e redução de doenças e infecções determinadas e perpetuadas pela pobreza, fome e iniquidades sociais, dentre elas a hanseníase^10^ ^)-(^ ^11^.

O conhecimento insuficiente das pessoas é a principal causa do medo e preconceito, embora a enfermidade tenha cura. Acredita-se que a adesão às orientações demanda ações educativas realizadas pelos profissionais no âmbito da Estratégia Saúde da Família (ESF), em especial o enfermeiro, e que devem ser iniciadas no momento do diagnóstico, para que o paciente seja informado e esclarecido sobre atividades de prevenção e autocuidado a serem praticadas por toda a vida^1^ ^),(^ ^12^.

Este estudo fornece informações importantes sobre a prática do autocuidado de pessoas com hanseníase, abrindo a possibilidade de os profissionais instrumentalizarem a prática no controle da doença de forma mais adequada, dando ênfase às orientações sobre o autocuidado, o que seguramente irá refletir na melhor qualidade de vida das pessoas afetadas. Assim, as contribuições para o serviço de saúde serão efetivas ao incentivar ações que previnam agravos, como as incapacidades físicas, o que reverbera em contribuições para qualidade de vida. Nessa perspectiva, este estudo teve por objetivo analisar práticas de autocuidado e fatores associados em pessoas com hanseníase na Atenção Primária à Saúde (APS).

## Métodos

### Tipo do estudo

Estudo transversal, reportado seguindo as recomendações do *Strengthening the Reporting of Observational studies in Epidemiology* (STROBE)^13^.

### Local de estudo

O estudo foi realizado nas Unidades Básicas de Saúde (UBS) do município de Campo Maior-PI, que possui população residente de 45.793 habitantes, localizado na região Norte do estado, a 82 km da capital, Teresina^14^. O município dispõe de 18 UBS com 21 equipes da ESF, sendo 15 na zona urbana e seis na zona rural. Todas as unidades participam do Programa Previne Brasil, que tem como principal objetivo promover a ampliação do acesso e a melhoria da qualidade da Atenção Primária à Saúde. 

### Período

A coleta de dados foi realizada nos meses de outubro a dezembro de 2023.

### População

A população foi composta pelo universo de pessoas afetadas por hanseníase e em acompanhamento na ESF do município de Campo Maior (n=145), cujos casos foram notificados no Sistema de Informação de Agravos de Notificação (SINAN), no período de janeiro de 2019 a dezembro de 2023. A escolha do intervalo de cinco anos considerou o tempo que o Ministério da Saúde (MS) preconiza para avaliação de contatos^1^. Do total de casos, três não foram localizados, seis foram a óbito por outras causas não relacionadas à hanseníase e três apresentavam sinais de demência face à idade avançada, finalizando em 133 o número de participantes do estudo. 

### Critérios de inclusão/exclusão

Foram incluídas pessoas em tratamento da hanseníase ou em fase de avaliação pós-tratamento, independentemente da idade e do sexo. Foram excluídas as pessoas com sinais de demência frente à idade avançada.

### Variáveis do estudo

A variável dependente foi a prática de autocuidado com os desfechos binários: insatisfatória e satisfatória. As variáveis independentes foram todas as demais, as quais foram classificadas como sociodemográficas (sexo, raça/cor, idade, situação conjugal, escolaridade, ocupação, renda mensal média, acesso a programas sociais, situação da moradia atual, familiares residentes na mesma casa e religião); clínicas/epidemiológicas (tempo do diagnóstico, dose de tratamento da Poliquimioterapia Única (PQT-U), classificação operacional, baciloscopia, forma clínica, grau de incapacidade física no diagnóstico, reações hansênicas, início do episódio reacional, úlceras plantares/palmares, doenças associadas e neurite); orientações recebidas; atitudes frente ao autocuidado em hanseníase (se observar e cuidar da face, das mãos e dos pés é necessário); e fatores limitantes do autocuidado. 

### Instrumento do estudo

Foi utilizado o instrumento Atitude e Prática de Autocuidado na Hanseníase: face, mãos e pés - APAHansen. Os itens do instrumento foram submetidos à validação de conteúdo, por um comitê composto por oito juízes. O IVC global do instrumento foi de 0,91 para clareza e 0,96 para relevância dos itens, evidenciando validade de conteúdo satisfatória. A intensidade da concordância entre os juízes foi medida por meio do Índice Kappa (K), obtendo-se valor ≥ 0,90, o que indica concordância excelente ou quase perfeita^15^. 

O APAHansen é composto por duas partes. A primeira contém dados sobre o perfil sociodemográfico e a segunda, aborda as orientações recebidas, as atitudes, as práticas e as limitações para o autocuidado. Quanto às atitudes de autocuidado, são seis questões e em cada uma das alternativas, cujas conotações são positivas, foi atribuído um valor numérico que varia de zero a dois, entendendo-se que zero corresponde à alternativa “Não”; um ponto a “Não deseja responder” e dois pontos, “Sim”, sendo que a atitude mais positiva totalizou 12 pontos, o que corresponde a 100%. Assim, a atitude foi classificada como segue: de 1 a 74% - Atitude negativa; de 75 a 100% - Atitude positiva^15^. 

A seção sobre práticas de autocuidado contém 46 questões divididas em três dimensões: 13 questões para a face, 11 para as mãos e 22 para os pés. Para cada dimensão, calculou-se a média aritmética das pontuações, possibilitando a classificação inicial das práticas de autocuidado dos participantes em três categorias: insatisfatórias (média de 1,0 a 2,5 pontos), precisa melhorar (média de 2,6 a 3,5 pontos) e satisfatórias (média de 3,6 a 5,0 pontos). Posteriormente, essas variáveis foram recategorizadas com o objetivo de facilitar as análises estatísticas, considerando-se que a categoria “precisa melhorar” ainda não representa um nível satisfatório de autocuidado. Dessa forma, as práticas de autocuidado foram dicotomizadas conforme os seguintes pontos de corte: insatisfatórias (média de 1,0 a 3,5 pontos) e satisfatórias (média de 3,6 a 5,0 pontos)^15^.

### Coleta de dados

A coleta de dados foi realizada de forma presencial, no período de outubro a dezembro de 2023, por meio da aplicação do formulário de forma individualizada e em local que garantisse conforto, sigilo e privacidade ao participante, após contato prévio, que ocorreu por meio de telefone ou presencialmente, nos momentos de atendimentos nas UBS. 

Após autorização da Secretaria Municipal de Saúde para utilização dos dados do SINAN e abordagem dos participantes do estudo nas UBS, a pesquisadora fez contato inicial com os enfermeiros das UBS. Nesse primeiro momento, foi discutida estratégias de operacionalização da coleta dos dados (datas, horários e acesso ao SINAN).

Dados sobre o perfil clínico e epidemiológico dos participantes foram obtidos diretamente da base de dados do SINAN, referentes ao tempo do diagnóstico, dose de tratamento com PQT-U, classificação operacional, baciloscopia, forma clínica, grau de incapacidade física no diagnóstico, reações hansênicas, início do episódio reacional, úlceras palmares/plantares, doenças associadas e neurite.

A anuência dos participantes para participar do estudo foi obtida por meio Termo de Consentimento Livre e Esclarecido (TCLE) e Termo de Assentimento Livre e Esclarecido (TALE). 

### Tratamento e análise dos dados

Os dados coletados foram exportados para o programa *Statistical Package for the Social Sciences* (SPSS) versão 26. Utilizou-se análise descritiva, com distribuição de frequências absoluta e percentual, medidas de posição e de dispersão. Na associação entre as variáveis categóricas e as práticas de autocuidado em hanseníase, utilizou-se o teste de qui-quadrado e/ou exato de Fisher, com correção de Yates. 

No estudo das práticas de autocuidado como variável resposta, utilizou-se a regressão logística, com razão de chance (*Odds Ratio* - OR) e respectivos intervalos de confiança, buscando explicar fatores ligados à prática de autocuidado insatisfatória da população do estudo. O critério para inclusão de variáveis no modelo logístico múltiplo foi a associação ao nível de 20% (p˂0,200) na análise bivariada e no modelo final, por sua vez, a significância estatística foi fixada em 5% (p˂0,05)^16^.

### Aspectos éticos

Esta pesquisa foi iniciada após autorização pela Secretaria Municipal de Saúde do município em estudo e aprovação pelo Comitê de Ética em Pesquisa (CEP) da Universidade Federal do Piauí (UFPI), sob parecer de número 6.323.688, aprovado em 26 de setembro de 2023.

## Resultados

Dentre os 133 participantes, 54,1% eram do sexo masculino e 75,2% se declararam pardos. A faixa etária predominante foi de 20 a 59 anos (62,4%), situação conjugal casado (60,9%), escolaridade compatível com ensino fundamental incompleto (51,1%), ocupação como aposentado (28,6%), renda entre um e dois salários-mínimos (48,9%), sem acesso a programas sociais (66,2%), com moradia própria (78,9%) e religião católica (79,7%) de acordo com a Tabela 1.

**Tabela 1:** Perfil sociodemográfico de pessoas afetadas por hanseníase e em acompanhamento na ESF (n* = 133). Campo Maior, PI, Brasil, 2023

Variáveis	n* (%)	IC-95%^†^	Média	Dp^‡^
Sexo				
Masculino	72 (54,1)	(45,7-62,4)		
Feminino	61 (45,9)	(37,6-54,3)		
Raça/cor				
Branca	23 (17,3)	(11,6-24,4)		
Parda	100 (75,2)	(66,6-81,3)		
Preta	10 (7,5)	(3,9-12,9)		
Faixa etária			49,2	17,8
≤ 19 anos	10 (7,5)	(3,9-12,9)		
20-59 anos	83 (62,4)	(54,0-70,3)		
≥60 anos	40 (30,1)	(22,8-38,2)		
Situação conjugal				
Casado	81 (60,9)	(30,4-58,8)		
Solteiro/Divorciado/Separado/Viúvo	54 (39,1)	(21,4-50,3)		
Escolaridade				
Sem escolaridade	13 (9,8)	(5,6-15,7)		
Ensino fundamental incompleto	68 (51,1)	(42,7-59,5)		
Ensino fundamental completo	06 (4,5)	(1,9-9,1)		
Ensino médio incompleto	12 (9,0)	(5,0-14,8)		
Ensino médio completo	27 (20,3)	(14,1-27,7)		
Ensino superior incompleto	01 (0,8)	(0,1-3,5)		
Ensino superior completo	06 (4,5)	(1,9-9,1)		
Ocupação				
Estudante	11 (8,3)	(4,5-13,9)		
Dona de casa	24 (18,0)	(12,2-25,2)		
Aposentado	38 (28,6)	(21,4-36,6)		
Desempregado	08 (6,0)	(2,9-11,0)		
Outros	52 (39,1)	(31,1-47,6)		
Renda mensal média				
<1 SM^§^	40 (30,1)	(22,8-38,2)		
1-2 SM^§^	65 (48,9)	(40,5-57,3)		
≥2 SM^§^	11 (8,3)	(3,4-12,0)		
Sem renda	17 (12,8)	(7,9-19,2)		
Acesso a programas sociais				
Sim	45 (33,8)	(26,2-42,2)		
Não	88 (66,2)	(57,8-73,8)		
Situação de moradia				
Casa própria	105 (78,9)	(71,4-85,2)		
Casa alugada	15 (11,3)	(6,7-17,5)		
Casa cedida	11 (8,3)	(4,5-13,9)		
Outros	02 (1,5)	(0,3-4,7)		
Pessoas que moram na casa			2,7	1,2
Religião				
Católica	106 (79,7)	(72,3-85,9)		
Evangélica	16 (12,0)	(7,3-18,4)		
Espírita	03 (2,3)	(0,6-5,9)		
Sem religião	05 (3,8)	(1,4-8,0)		
Outros	03 (2,3)	(0,6-5,9)		

*n = Tamanho da amostra; ^†^IC-95% = Intervalo de Confiança para proporção; ^‡^Dp = Desvio-Padrão; ^§^SM = Salário-mínimo vigente = R$ 1.320,00, Brasil, 2023

A maioria (98,5%) afirmou receber orientações dos profissionais sobre práticas de autocuidado na UBS, sendo o enfermeiro o profissional citado por 99,2% dos entrevistados. 

A atitude da população investigada sobre o autocuidado foi classificada como positiva em 98,5% dos casos e negativa em 1,5%. Na classificação das práticas de autocuidado, foram investigados três segmentos: face, mãos e pés, sendo que em nenhum deles observou-se o predomínio de práticas satisfatórias (Tabela 2).

**Tabela 2:** Classificação das práticas de autocuidado da amostra do estudo relativas aos segmentos face, mãos e pés (n* = 133). Campo Maior, PI, Brasil, 2023

Variáveis	n* (%)	IC-95%^†^	Média	Dp^‡^
Prática de autocuidado-face			2,6	0,59
Insatisfatórias	66 (49,6)	(41,2-58,1)		
Precisa melhorar	61 (45,9)	(37,6-54,3)		
Satisfatórias	06 (4,5)	(1,9-9,1)		
Prática de autocuidado-mãos			2,7	0,67
Insatisfatórias	57 (42,9)	(34,7-51,3)		
Precisa melhorar	59 (44,4)	(36,1-52,8)		
Satisfatórias	17 (12,8)	(7,9-19,2)		
Prática de autocuidado-pés			2,8	0,48
Insatisfatórias	39 (29,3)	(22,1-37,4)		
Precisa melhorar	87 (65,4)	(57,1-73,1)		
Satisfatórias	07 (5,3)	(2,4-10,1)		

*n = Tamanho da amostra; ^†^IC-95% = Intervalo de Confiança para proporção; ^‡^Dp = Desvio-Padrão

Dentre os casos investigados, 95,5% apresentavam a classificação operacional multibacilar, com baciloscopia negativa para 70,7%, forma clínica dimorfa (90,2%) e GIF 1 na ocasião do diagnóstico em 62,4%. A maioria nunca apresentou reação hansênica (80,5%) e dentre os que apresentaram, 18% foi do tipo 1, porém sem neurite (88%), e com início do episódio reacional observado no momento do diagnóstico. As úlceras plantares e palmares foram ausentes em 88% dos investigados. As doenças associadas foram Diabetes Mellitus (DM) (15,8%) e Hipertensão Arterial Sistêmica (HAS) (30,8%). Outras doenças incluíram hipertireoidismo, ansiedade, dislipidemia e cardiopatias (Tabela 3).

**Tabela 3:** Caracterização clínica dos casos de hanseníase do estudo (n* = 133). Campo Maior, PI, Brasil, 2023

Dados clínicos	n* (%)	IC-95%^†^
Classificação operacional		
Paucibacilar	06 (4,5)	(1,9-9,1)
Multibacilar	127 (95,5)	(90,9-98,1)
Baciloscopia		
Positivo	12 (9,0)	(5,0-14,8)
Negativo	94 (70,7)	(62,6-77,9)
Não realizada	27 (20,3)	(14,1-27,7)
Forma clínica		
Indeterminada	05 (3,8)	(1,4-8,0)
Tuberculoide	02 (1,5)	(0,3-4,7)
Dimorfa	120 (90,2)	(84,3-94,4)
Virchowiana	06 (4,5)	(1,9-9,1)
GIF^‡^ no diagnóstico		
GIF^‡^ 0	39 (29,3)	(22,1-37,4)
GIF^‡^ 1	83 (62,4)	(54,0-70,3)
GIF^‡^ 2	11 (8,3)	(4,5-13,9)
Reação hansênica		
Tipo I	24 (18,0)	(12,2-25,2)
Tipo II	01 (0,8)	(0,1-3,5)
Nunca teve	107 (80,5)	(73,1-86,5)
Já teve em outro momento	01 (0,8)	(0,1-3,5)
Início do episódio reacional		
Diagnóstico	13 (9,8)	(5,6-15,7)
Tratamento	08 (6,0)	(2,9-11,0)
Pós-alta	05 (3,8)	(1,4-8,0)
Não se aplica	107 (80,5)	(73,1-86,5)
Úlceras plantares/palmares		
Atual	07 (5,3)	(2,4-10,1)
Prévio	01 (0,8)	(0,1-3,5)
Nunca teve	117 (88,0)	(81,6-92,7)
Sem registro	08 (6,0)	(2,9-11,0)
Doenças associadas		
Diabetes Mellitus		
Não	112 (84,2)	(77,3-89,6)
Sim	21 (15,8)	(10,4-22,7)
Hipertensão arterial		
Não	92 (69,2)	(61,0-76,5)
Sim	41 (30,8)	(23,5-39,0)
Outros		
Não	46 (34,6)	(26,9-42,9)
Sim	87 (65,4)	(57,1-73,1)
Neurite		
Atual	13 (9,8)	(5,6-15,7)
Prévio	02 (1,5)	(0,3-4,7)
Não se aplica	117 (88,0)	(81,6-92,7)
Sem registro	01 (0,8)	(0,1-3,5)

*n = Tamanho da amostra; ^†^IC-95% = Intervalo de Confiança para proporção; ^‡^GIF = Grau de Incapacidade Física

No modelo de regressão múltipla, apenas duas variáveis se mantiveram estatisticamente associadas às práticas de autocuidado insatisfatórias: ter úlceras plantares ou palmares associou-se à redução de 10% nas chances de apresentar autocuidado insatisfatório (ORa = 0,90; p = 0,05) e a presença de neurites reduziu as chances em 59% (ORa = 0,41; p = 0,04) (Tabela 4).

**Tabela 4:** Modelos logísticos da prática de autocuidado insatisfatória das pessoas com hanseníase (n* = 133). Campo Maior, PI, Brasil, 2023

Variáveis	Análise bivariada				Análise múltipla			
	ORb*	IC 95%^†^		p- valor^||^	ORa^¶^	IC 95%^†^		p- valor^||^
		LI^‡^	LS^§^			LI^‡^	LS^§^	
Dados sociodemográficos								
Sexo				0,23				
Masculino	1,57	0,74	3,33					
Feminino	1							
Raça/cor								
Branca	1,52	0,32	7,14	0,59				
Parda	1,71	0,45	6,53	0,43				
Preta	1							
Situação conjugal								
Casado/União estável	0,92	0,44	2,07	0,92				
Divorciado/Solteiro/Viúvo	1							
Escolaridade								
Sem escolaridade	1,66	0,19	14,05	0,63				
Ensino fundamental	1,04	0,17	6,09	0,96				
Ensino médio	1,50	0,23	9,46	0,66				
Ensino superior	1							
Ocupação								
Estudante	0,98	0,22	4,23	0,98				
Dona de casa	0,89	0,30	2,61	0,89				
Aposentado	0,70	0,28	1,75	0,70				
Desempregado	1,10	0,19	6,13	1,10				
Outros	1							
Renda mensal média								
<1 SM**	0,50	0,12	2,06	0,33				
1-2 SM**	0,44	0,11	1,73	0,24				
Sem renda	1							
Classificação operacional								
Paucibacilar	-			0,99				
Multibacilar	1							
Forma clínica								
Indeterminada	2,00	0,12	31,97	0,62				
Tuberculoide	-			0,99				
Dimorfa	1,16	0,20	6,65	0,86				
Virchowiana	1							
GIF^††^ no diagnóstico								
Não	1							
Sim	1,01	0,48	2,48	0,81				
Reação hansênica								
Não	1				1			
Sim	0,36	0,14	0,88	0,02	-			0,99
Início do episódio reacional								
Diagnóstico	0,27	0,08	0,89	0,03	-			1,00
Tratamento	0,32	0,07	1,375	0,12	-			1,00
Pós-alta	0,48	0,07	3,04	0,43	-			1,00
Não se aplica	1				1			
Úlceras plantares/palmares								
Não					1			1
Sim	0,38	0,09	1,64	0,09	0,90	0,16	4,80	0,05
Diabetes								
Não	1							
Sim	1,04	0,37	2,92	0,93				
Hipertensão arterial								
Não	1							
Sim	0,84	0,38	1,88	0,68				
Outros								
Não	1							
Sim	1,27	0,58	2,75	0,54				
Neurite								
Não	1				1			
Sim	0,31	0,10	0,93	0,03	0,41	0,12	1,37	0,04
Atitude de Autocuidado								
Atitude Negativa	1,51	0,31	1,11	0,99				
Atitude Positiva								

*ORb = Razão de chance bruta; ^†^IC-95% = Intervalo de Confiança para proporção; ^‡^LI = Limite inferior; ^§^LS = Limite superior; ^||^p-valor; ^¶^ORa = Razão de chance ajustada; **SM = Salário-mínimo vigente = R$ 1.320,00, Brasil, 2023; ^††^GIF = Grau de Incapacidade Física

## Discussão

Na população estudada, o sexo masculino representou a maioria dos casos novos com hanseníase multibacilar, especialmente na faixa etária de 20 a 59 anos, com ensino fundamental incompleto e renda familiar baixa. A hanseníase atinge pessoas suscetíveis e em países endêmicos, principalmente os da América Latina, onde fatores como baixo nível socioeconômico, desnutrição, população rural dispersa e acesso limitado e inadequado a serviços de saúde para diagnóstico e tratamento, entre outras características, desempenham importante influência na cadeia epidemiológica de transmissão e ampliam a prevalência oculta da doença. Além disso, a condição econômica pode ser um possível obstáculo para a adesão e finalização do tratamento^17^ ^)-(^ ^22^.

A escolaridade também é um fator determinante para a eficácia das ações de educação em saúde, pois atua como um mediador no processo de compreensão da doença e na adesão ao tratamento. Compreende-se que indivíduos com maior grau de instrução tendem a apresentar maior facilidade no domínio de conceitos relacionados à prevenção e identificação precoce de sinais e sintomas, o que, consequentemente, favorece as práticas de autocuidado e reduz o risco de incapacidades físicas. Um estudo randomizado com 635 pessoas afetadas por hanseníase na Nigéria concluiu que a escolaridade, embora possa estar relacionada à melhor compreensão da autoimagem e do enfrentamento, pode não proteger os indivíduos do estigma vivenciado na comunidade. Essa constatação reforça a necessidade de programas e campanhas educacionais aprimoradas para abordar concepções errôneas e melhorar a conscientização sobre a hanseníase^23^.

Um achado deste estudo que chamou a atenção refere-se à proporção de casos multibacilares com grau 1 de incapacidade física, o que reforça a prevalência oculta da doença associada a diagnóstico tardio e/ou preparo insuficiente dos profissionais da APS para o diagnóstico correto e oportuno dos casos^24^. Uma pesquisa de base populacional realizada na China apontou que, apesar do progresso na redução da prevalência de incapacidades físicas relacionadas à hanseníase, entre 1990 e 2020, a proporção de casos com incapacidades, entre os afetados pela doença, permanece alta, o que indica detecção em estágios mais avançados e transmissão comunitária ainda persistente^25^. É fundamental qualificar e ampliar as ações de detecção precoce, prevenção e tratamento oportuno, considerando ser a forma clínica com maior importância epidemiológica, pois elimina maior número de bacilos, o que contribui para a manutenção da cadeia de transmissão^17^.

A baciloscopia foi realizada na maioria dos casos, obtendo-se resultado negativo. Trata-se de um exame laboratorial complementar ao diagnóstico clínico, cujo objetivo é detectar a presença do *M. leprae* em esfregaços de raspado intradérmico e estimar a carga bacilar apresentada pelo paciente. Quando positiva, indica caso multibacilar; quando negativa, não permite descartar o diagnóstico da doença e sua realização não é obrigatória para o diagnóstico clínico. Além disso, o exame auxilia no monitoramento do tratamento, possibilitando comparar o índice bacilar no início e ao término do mesmo^1^ ^),(^ ^26^.

Destaca-se que as reações hansênicas foram ausentes na maioria dos participantes, porém, nos que as apresentaram, foram do tipo I. O aparecimento de reações é uma das principais dificuldades no manejo clínico das pessoas com hanseníase, pois são episódios agudos e inflamatórios que podem ocorrer antes, durante e após a poliquimioterapia, e causar incapacidades físicas irreversíveis nos nervos quando não reconhecidas e tratadas de forma adequada^27^.

A atitude das pessoas afetadas por hanseníase frente ao autocuidado foi em sua expressiva maioria positiva. Por outro lado, predominaram as práticas insatisfatórias de autocuidado. No geral, espera-se que uma atitude favorável seja precursora de uma prática saudável. Contudo, nem sempre há coerência entre o que as pessoas acreditam e suas ações práticas, o que pode ser compreendido como uma dissonância entre atitude e comportamento. 

De acordo com a teoria da consistência cognitiva, uma atitude é provavelmente precursora de um padrão de comportamento. Contudo, desde 1934, La Pierre já havia se referido às inconsistências entre atitudes e práticas, apontando disparidades entre o que se diz e o que faz. Reforçando essa ideia, afirma-se que a incoerência cognitiva está sempre presente para a maioria das pessoas, na maior parte do tempo e para todas as pessoas uma parte do tempo. Desse modo, esse enfoque foi impugnado do ponto de vista empírico e teórico^28^ ^)-(^ ^29^.

Por outro lado, o enfoque da Consistência Contingente analisa a consistência entre atitude e prática do sujeito em relação ao objeto e às circunstâncias situacionais que o rodeiam, apontando a importância da rede de apoio ou apoios estruturais, considerando dois tipos de indivíduos: os que mantêm uma consistência permanente entre atitudes e práticas, independentemente dos apoios e os ambivalentes, que alteram o comportamento em função de apoios ou de pressões momentâneas^29^ ^)-(^ ^30^.

Em suma, conforme essa teoria da Consistência Contingente, para que não haja dissonâncias entre atitudes e práticas, faz-se necessário considerar a situação em que o objeto se encontra, e somente a partir do controle dos fatores circunstanciais negativos o sujeito pode alcançar uma atitude positiva, que lhe propicie melhores níveis de saúde. 

Nesta perspectiva, é fundamental a execução do autocuidado como fator primordial na prevenção de incapacidades e na responsabilização da pessoa para a manutenção da sua saúde^9^ ^),(^ ^31^, porém existem os contextos de vulnerabilidade social, em que barreiras estruturais e falta de insumos dificultam a materialização das atitudes positivas em práticas consistentes de autocuidado, podendo este ser influenciado por habilidades, limitações, condições culturais, sociais e econômicas que interferem de modo direto na saúde de quem o realiza^32^. No estudo realizado na província de Jiangsu, na China, a capacidade de autocuidado dos grupos tratados em serviços de saúde e em domicílio foi influenciada pela habilidade de autocuidado e pelo letramento em saúde^33^.

Desse modo, em saúde não é suficiente conhecer apenas as atitudes e práticas do indivíduo em relação a determinada situação, mas as contingências em que está inserido. Assim, embora a pessoa afetada possa ser favorável ao autocuidado, ela pode não compreender exatamente como fazê-lo ou qual o fundamento da sua realização**.** Além disso, a ausência de suporte contínuo e o desconhecimento técnico sobre os cuidados necessários reforçam essa dissociação entre atitude e prática, evidenciando lacunas na educação em saúde^34^.

Considerando que o estigma pode ser uma barreira para a realização do autocuidado, a literatura evidencia que o apoio entre pares e o apoio psicossocial, por meio de grupos de autoajuda, contribuem significativamente para sua redução entre pessoas afetadas pela hanseníase e a comunidade na qual estão inseridas. Esses grupos podem desempenhar um papel fundamental nas intervenções de redução do estigma sobre as doenças tropicais negligenciadas, contribuindo para um diagnóstico mais rápido, maior adesão à terapia e às práticas de autocuidado^23^ ^),(^ ^35^. 

A hanseníase segue como um grande desafio para a saúde pública nacional e internacional, considerando-se tratar de uma doença que afeta não somente a pele, mas também os nervos periféricos, com forte tendência a gerar deformidades e incapacidades, bem como processos inflamatórios (neurites)^36^ ^)-(^ ^39^. Destaca-se que as práticas insatisfatórias eram reduzidas quando os participantes apresentavam úlceras plantares ou palmares, ou ainda neurites. As demandas de autocuidado estão diretamente relacionadas aos olhos, face, membros superiores e inferiores, especialmente mãos e pés, considerando-se que são os principais sítios de acometimento cutâneo e neural^32^.

A neuropatia periférica é uma das principais manifestações da doença, pois compromete as funções sensoriais, motoras e autonômicas, o que resulta em deformidades visíveis e comprometimento da função neural^1^ ^),(^ ^40^. Entre as deformidades físicas mais comuns, a úlcera plantar é uma das principais. A região plantar é o local mais acometido por úlceras, pelo fato de as pessoas não perceberem traumas físicos como ferimentos e queimaduras, e as feridas ficam continuamente expostas ao impacto da locomoção e ao contato, por diminuição ou perda da sensibilidade. O atraso no diagnóstico é a principal razão para o risco de desenvolvimento de incapacidades, como as úlceras^41^ ^)-(^ ^42^.

O enfermeiro, como um dos profissionais integrantes da equipe da APS, torna-se elemento-chave na execução das ações propostas pelo Programa Nacional de Controle e Eliminação da Hanseníase (PNCEH), atuando no cuidado integral dos pacientes, desde o diagnóstico até a conclusão do tratamento, além de orientar as práticas de autocuidado^43^ ^)-(^ ^44^. Destaca-se que o envolvimento de profissionais capacitados e com comunicação eficaz é facilitador do reconhecimento precoce dos sinais e sintomas, do acesso aos serviços de saúde, do incentivo ao tratamento e da motivação para a prática do autocuidado com vistas a prevenir incapacidades relacionadas à hanseníase^45^ ^)-(^ ^46^.

Contribuindo com a prerrogativa anterior, a investigação realizada nos distritos de Dhanusa e Mahottari, Nepal, para avaliar o conhecimento e as habilidades de 31 profissionais de saúde locais, concluiu que o desempenho mediano geral dos cuidados com a hanseníase foi baixo (53%) e poderia ser melhorado por meio de treinamento baseado em evidências, acompanhamento presencial, monitoramento e supervisão, com o objetivo de facilitar a eliminação da transmissão da hanseníase. Entretanto, o estudo observou que o aconselhamento realizado por profissionais de saúde especializados melhorou significativamente as crenças e as práticas positivas dos pacientes em relação ao autocuidado durante a realização do estudo^47^.

A hanseníase, sem intervenção precoce, pode causar danos nervosos irreversíveis, comprometimento e incapacidade, implicando não apenas em um problema de saúde, mas também em um problema social. Evidencia-se o autocuidado como uma das principais estratégias na prevenção, monitoramento, gerenciamento e controle de incapacidades**,** sendo o indivíduo responsável pela manutenção e promoção de sua própria vida, saúde e bem-estar^44^ ^),(^ ^48^.

O potencial incapacitante causado pela doença é bastante significativo e a avaliação das incapacidades é importante para a educação e promoção do autocuidado. A prevenção é feita por meio de procedimentos e exercícios que a própria pessoa, devidamente orientada e capacitada, deverá realizar regularmente no próprio domicílio durante o tratamento e após a alta. É fundamental uma mudança de comportamento, bem como a inserção do autocuidado na rotina das pessoas afetadas^49^.

A utilização de Tecnologias Educacionais contribui para a melhora significativa na adesão ao autocuidado com a face, mãos e pés. Além disso, melhora os índices de independência durante a realização das atividades de vida diária, aumenta a frequência das práticas de autocuidado físico e mental e a adesão ao tratamento da hanseníase^50^. 

Uma das limitações identificadas diz respeito à abordagem de pessoas que tiveram hanseníase entre 2019 e 2023, isto é, em um intervalo de até cinco anos. Nesse período, é natural que tenham ocorrido mudanças de endereço e óbitos. O tempo também foi um fator que pode ter contribuído para possíveis vieses de memória. Ademais, a escassez de estudos sobre práticas de autocuidado em hanseníase implicou em algumas dificuldades nas comparações; contudo, não trouxe prejuízos ao estudo.

Este estudo traz contribuições valiosas para o campo do autocuidado em hanseníase, sobretudo ao reforçar a necessidade de intervenções que transcendam o aconselhamento verbal, com foco em oficinas demonstrativas e no acompanhamento longitudinal das habilidades de autocuidado que estão sendo desenvolvidas. 

Também contribui ao evidenciar que o foco das ações programáticas deve ser redirecionado para o paciente sem incapacidades físicas ou sem lesões cutâneas aparentes, com o intuito de romper o ciclo de agravamento que pode levar a danos irreversíveis, promover o empoderamento frente à prevenção dessas incapacidades por meio do autocuidado e possibilitar o acesso aos insumos necessários para realizá-lo de forma satisfatória. 

## Conclusão

Os resultados do estudo revelam que a maioria dos participantes afetados pela hanseníase encontra-se inserida em um contexto sociodemográfico e econômico de desigualdades sociais, no qual prevalecem a baixa renda e o baixo nível de escolaridade. Embora não tenha sido observada uma associação estatisticamente significativa entre os dados sociodemográficos e as práticas de autocuidado com a face, mãos e pés, pode haver uma importância clínica. Pressupõe-se que a adoção e o desenvolvimento dessas atividades sejam influenciados por outros fatores, como aqueles relacionados à equipe de saúde e a variáveis subjetivas. 

A atitude frente ao autocuidado foi positiva na expressiva maioria dos participantes; porém, predominaram as práticas de autocuidado insatisfatórias, as quais eram significativamente reduzidas na presença de úlceras plantares ou palmares, ou ainda de neurites.

As ações de educação em saúde são estratégias primordiais no enfrentamento da hanseníase que devem ser implementadas pelos profissionais das equipes da APS, que têm importante papel no cuidado integral dos usuários, tanto na identificação precoce, avaliação e prevenção de complicações, adesão ao tratamento e promoção da recuperação, quanto no acompanhamento das demandas de autocuidado. Para tanto, faz-se necessário que esses profissionais sejam adequadamente qualificados.

A criação e implementação de grupos de ajuda mútua é uma estratégia que possibilita às pessoas afetadas pela hanseníase um espaço para compartilhar experiências e saberes, desenvolver suas habilidades, ampliar o conhecimento acerca dos cuidados necessários, com foco na prevenção de ulcerações, sequelas e incapacidades.

## Data Availability

Todos os dados gerados ou analisados durante este estudo estão incluídos neste artigo publicado.

# Translation: Prácticas de autocuidado y factores asociados en personas con enfermedad de Hansen en la Atención Primaria*

**DOI:** 10.1590/1518-8345.7921.4960

**Authors:** Michelle Santos Macêdo, Nanielle Silva Barbosa, Olívia Dias de Araújo, Fernanda Lorrany Silva, Lídya Tolstenko Nogueira, Telma Maria Evangelista de Araújo

**Affiliations:** 1 Universidade Federal do Piauí, Departamento de Pós-Graduação em Enfermagem, Teresina, PI, Brasil.; 2 Becaria de la Coordenação de Aperfeiçoamento de Pessoal de Nível Superior (CAPES), Brasil.

**Keywords:** Práctica, Autocuidado, Lepra, Atención Primaria de Salud, Enfermería, Diagnóstico Temprano

## Abstract

**Objetivo:**

analizar las prácticas de autocuidado y los factores asociados en personas con enfermedad de Hansen en la Atención Primaria de Salud.

**Método:**

estudio transversal realizado en Unidades Básicas de Salud con 133 casos de enfermedad de Hansen. Se utilizó el instrumento Actitud y Práctica de Autocuidado en la enfermedad de Hansen - Cara, Manos y Pies. Para analizar la asociación entre las variables se utilizaron la prueba de chi-cuadrado y/o la prueba exacta de Fisher, con corrección de Yates. Para el análisis de las prácticas de autocuidado se empleó regresión logística, con *odds ratio* y sus respectivos intervalos de confianza.

**Resultados:**

predominó el sexo masculino (54,1%), rango de edad de 20 a 59 años (62,4%), casados (60,9%), ingreso de uno a dos salarios mínimos (48,9%). La actitud frente a el autocuidado fue positiva (98,5%). Entre los participantes, solo el 4,5% realizaban prácticas de autocuidado relacionadas con la cara, las manos (12,8%) y los pies (5,3%). Tener úlceras plantares o palmares y neuritis se asoció con una menor probabilidad de autocuidado insatisfactorio.

**Conclusión:**

la actitud frente el autocuidado fue positiva en la gran mayoría de los participantes, mostrando disonancia con las prácticas de autocuidado, que fueron predominantemente insatisfactorias. El autocuidado en la enfermedad de Hansen es esencial para la prevención de secuelas y requiere que los profesionales de la salud desarrollen orientación y monitoreo continuos, con el objetivo de mejorar la calidad de vida.

## Destacados

**(1)** El impacto de la enfermedad de Hansen y su influencia en el autocuidado de la persona.

**(2)** La práctica del autocuidado en el mantenimiento de la integridad funcional de la persona.

**(3)** Las incapacidades producidas por la enfermedad pueden provocar limitaciones.

**(4)** La importancia del autocuidado en la prevención de incapacidades físicas.

**(5)** Comprensión de las personas afectadas por la enfermedad sobre el autocuidado.

## Introducción

La enfermedad de Hansen es una de las enfermedades más antiguas de la humanidad, originada en África Occidental hace unos 100.000 años y propagándose por todo el mundo por medio de las personas que se desplazaban en las rutas comerciales y por el colonialismo. Afecta principalmente a los nervios periféricos y la piel, pero también puede afectar sistémicamente a otros órganos^1^ ^)-(^ ^3^.

El número de casos nuevos a nivel mundial en 2022 fue de 174.087, lo que corresponde a una tasa de detección de 21,8 casos por millón de habitantes. India, Brasil e Indonesia reportaron más de 10 mil casos nuevos de enfermedad de Hansen cada uno y, en esta clasificación, estos países, incluido Brasil, siguen siendo prioritarios para la Organización Mundial de la Salud (OMS) debido al número de casos nuevos^2^.

Brasil es un país con una alta carga de enfermedad de Hansen y no ha alcanzado las metas de eliminación establecidas por la OMS, incluido el diagnóstico precoz^3^. En esta perspectiva, el Grado de Incapacidad Física (GIF) es un indicador epidemiológico utilizado en la evaluación del programa de vigilancia de la enfermedad y determina la precocidad del diagnóstico^4^ ^)-(^ ^5^.

Las discapacidades físicas adquiridas por el diagnóstico tardío de enfermedad de Hansen, principalmente GIF 2, afectan el cuerpo, la vida familiar y social. El autocuidado es la principal estrategia, tanto a nivel nacional como mundial, que debe ser ampliamente, reforzada desde el diagnóstico, con base en procedimientos, técnicas y ejercicios que minimicen y/o prevengan los efectos relacionados con los ojos, la cara y las extremidades superiores e inferiores, considerándose las principales zonas afectadas por la enfermedad^5^ ^)-(^ ^8^.

Las incapacidades y deformidades causadas por la enfermedad de Hansen pueden causar limitaciones, interferir con la capacidad laboral, social y familiar, y generar problemas psicológicos relacionados con el estigma y los prejuicios que pueden aislarlos de la interacción social^9^. Por lo tanto, es importante conocer el nivel de comprensión de las personas afectadas sobre el autocuidado, para poder implementar acciones que prevengan y/o minimicen las discapacidades.

La enfermedad de Hansen excluye a la persona de su comunidad debido al estigma y los prejuicios que aún persisten, además de la falta de inversión en investigación y desarrollo de nuevas tecnologías para su tratamiento y cura. Ante este problema, en 2024 se lanzó en el país el Programa Brasil Saludable - Unir para Cuidar, con la finalidad de promover acciones intersectoriales que contribuyan a la eliminación y reducción de enfermedades e infecciones determinadas y perpetuadas por la pobreza, el hambre y las desigualdades sociales, incluida la enfermedad de Hansen^10^ ^)-(^ ^11^.

La falta de conocimiento de las personas es la principal causa de miedo y prejuicio, a pesar de que la enfermedad es curable. Se cree que la adherencia a las orientaciones requiere acciones educativas realizadas por los profesionales en el ámbito de la Estrategia de Salud Familiar (ESF), especialmente el personal de enfermería, y que estas acciones deben comenzar desde el momento del diagnóstico, para que el paciente esté informado y orientado sobre las actividades de prevención y autocuidado que debe practicarse durante toda la vida^1^ ^),(^ ^12^.

Este estudio proporciona información importante sobre las prácticas de autocuidado de personas con enfermedad de Hansen, abriendo la posibilidad de que los profesionales fortalezcan esta práctica en el control de la enfermedad de forma más adecuada, enfatizando la orientación sobre el autocuidado, lo que sin duda se reflejará en una mejor calidad de vida para las personas afectadas. Por lo tanto, las contribuciones al servicio de salud serán efectivas al incentivar acciones que prevengan complicaciones, como incapacidades físicas, lo que repercute en mejoras en la calidad de vida. Desde esta perspectiva, este estudio tuvo como objetivo analizar las prácticas de autocuidado y los factores asociados en personas con enfermedad de Hansen en Atención Primaria de Salud (APS).

## Métodos

### Tipo de estudio

Estudio transversal, reportado siguiendo las recomendaciones de *Strengthening the Reporting of OBservational studies in Epidemiology* (STROBE)^13^.

### Lugar del estudio

El estudio se realizó en las Unidades Básicas de Salud (UBS) del municipio de Campo Maior-PI, con una población residente de 45.793 habitantes y ubicado en la región Norte del estado, a 82 km de la capital, Teresina^14^. El municipio cuenta con 18 UBS con 21 equipos de la ESF, 15 en el área urbana y seis en el área rural. Todas las unidades participan del Programa Previne Brasil, cuyo principal objetivo es promover la ampliación del acceso y la mejora de la calidad de la Atención Primaria de Salud.

### Período

La recolección de datos fue realizada entre octubre y diciembre de 2023.

### Población

La población estuvo compuesta por el universo de personas afectadas por la enfermedad de Hansen y en acompañamiento en la ESF en el municipio de Campo Maior (n=145), cuyos casos fueron notificados en el Sistema de Información de Enfermedades de Notificación (SINAN) entre enero de 2019 y diciembre de 2023. La elección del intervalo de cinco años consideró el marco temporal recomendado por el Ministerio de Salud (MS) para el rastreo de contactos^1^. Del total de casos, tres no pudieron ser localizados, seis fallecieron por causas no relacionadas con la enfermedad de Hansen y tres mostraron signos de demencia debido a la edad avanzada, finalizando en 133 el número de participantes del estudio.

### Criterios de inclusión/exclusión

Fueron incluidas las personas en tratamiento para la enfermedad de Hansen o en la fase de evaluación posterior al tratamiento, independientemente de su edad y sexo. Fueron excluidas las personas con signos de demencia debido a la edad avanzada.

### Variables de estudio

La variable dependiente fue la práctica de autocuidado, con resultados binarios: insatisfactorio y satisfactorio. Las variables independientes fueron todas las demás, que se clasificaron como sociodemográficas (sexo, raza/color, edad, estado civil, escolaridad, ocupación, ingreso mensual medio, acceso a programas sociales, situación actual de la vivienda, familiares que residen en la misma casa y religión); clínico/epidemiológicas [tiempo desde el diagnóstico, dosis de tratamiento con Poliquimioterapia Única (PQT-U), clasificación operacional, baciloscopia, forma clínica, grado de incapacidad física al diagnóstico, reacciones hansénicas, inicio del episodio reaccional, úlceras plantares/palmares, enfermedades asociadas y neuritis]; orientación recibida; actitudes hacia el autocuidado en la enfermedad de Hansen (si observar y cuidar la cara, las manos y los pies es necesario); y factores limitantes del autocuidado.

### Instrumento de estudio

Fue utilizado el instrumento Actitud y Práctica de Autocuidado en la enfermedad de Hansen: cara, manos y pies - APAHansen. Los ítems del instrumento fueron sometidos a validación de contenido por un comité compuesto por ocho jueces. El IVC global del instrumento fue de 0,91 para claridad y de 0,96 para relevancia de los ítems, evidenciando una validez de contenido satisfactoria. La intensidad de la concordancia entre los jueces fue medida mediante el Índice Kappa (K), obteniéndose un valor ≥ 0,90, lo que indica concordancia excelente o casi perfecta^15^.

El APAHansen es compuesto de dos partes. La primera contiene datos sobre el perfil sociodemográfico y la segunda aborda la orientación recibida, las actitudes, las prácticas y las limitaciones para el autocuidado. Respecto a las actitudes de autocuidado, existen seis preguntas a cada una de las alternativas, con connotaciones positivas, fue atribuido un valor numérico que varía de cero a dos, entendiéndose que cero corresponde a “No”; un punto a “No desea responder”; y dos puntos a “Sí”, siendo que la actitud más positiva totalizo 12 puntos, lo que corresponde al 100%. Por lo tanto, la actitud fue clasificada de la siguiente manera: 1 a 74% - Actitud negativa; 75 a 100% - Actitud positiva^15^.

La sección sobre prácticas de autocuidado contiene 46 preguntas divididas en tres dimensiones: 13 preguntas para la cara, 11 para las manos y 22 para los pies. Para cada dimensión, se calculó la media aritmética de las puntuaciones, posibilitando la clasificación inicial de las prácticas de autocuidado de los participantes en tres categorías: insatisfactorias (media de 1,0 a 2,5 puntos), necesita mejorar (media de 2,6 a 3,5 puntos) y satisfactorio (media de 3,6 a 5,0 puntos). Posteriormente, estas variables se recategorizaron con el objetivo de facilitar los análisis estadísticos, considerándose que la categoría “necesita mejorar” aún no representa un nivel satisfactorio de autocuidado. Por lo tanto, las prácticas de autocuidado fueron dicotomizadas conforme a los siguientes puntos de corte: insatisfactorias (media de 1,0 a 3,5 puntos) y satisfactorias (media de 3,6 a 5,0 puntos)^15^.

### Recolección de datos

La recolección de datos fue realizada de forma presencial, en el periodo de octubre a diciembre de 2023, mediante la aplicación del formulario de forma individualizada y en un lugar que garantizara comodidad, confidencialidad y privacidad al participante, después de un contacto previo, ocurrido vía telefónica o presencialmente, durante los atendimientos en las UBS. 

Tras obtener la autorización de la Secretaría Municipal de Salud para utilizar los datos del SINAN y el abordaje de los participantes del estudio en las UBS, la investigadora estableció contacto inicial con los enfermeros de las UBS. En esta primera etapa, fueron discutidas las estrategias de operacionalización de la recolección de datos (fechas, horarios y acceso al SINAN).

Los datos del perfil clínico y epidemiológico de los participantes se obtuvieron directamente de la base de datos del SINAN, referente al momento del diagnóstico, dosis de tratamiento con PQT-U, clasificación operacional, baciloscopia, forma clínica, grado de incapacidad física al diagnóstico, reacciones hansénicas, inicio del episodio reaccional, úlceras palmares/plantar, enfermedades asociadas y neuritis.

La anuencia de los participantes para participar en el estudio fue obtenida a través de un Término de Consentimiento Libre e Informado (TCLI) y un Término de Asentimiento Libre e Informado (TALI).

### Tratamiento y análisis de los datos

Los datos recopilados fueron exportados al programa *Statistical Package for the Social Sciences* (SPSS), versión 26. Se utilizó un análisis descriptivo con distribuciones de frecuencia absoluta y porcentual, medidas de posición y dispersión. En la asociación entre variables categóricas y prácticas de autocuidado en la enfermedad de Hansen se utilizó la prueba de chi- cuadrado y/o la prueba exacta de Fisher, con corrección de Yates. 

En el estudio de las prácticas de autocuidado como variable respuesta se utilizó la regresión logística, con razón de probabilidades (*Odds Ratio* - OR) y sus respectivos intervalos de confianza, buscando explicar los factores vinculados a prácticas de autocuidado insatisfactorias en la población de estudio. El criterio para la inclusión de variables en el modelo logístico múltiple fue la asociación al 20% (p˂0,200) en el análisis bivariado y, en el modelo final, la significancia estadística se fijó en el 5% (p˂0,05)^16^.

### Aspectos éticos

Esta investigación se inició después de la autorización de la Secretaría Municipal de Salud del municipio en estudio y aprobación del Comité de Ética en Investigación (CEI) de la Universidad Federal de Piauí (UFPI), bajo dictamen número 6.323.688, aprobado el 26 de septiembre de 2023.

## Resultados

Entre los 133 participantes, el 54,1% eran del sexo masculino y el 75,2% se declararon de raza parda. El rango de edad predominante fue de 20 a 59 años (62,4%), estado civil casado (60,9%), escolaridad compatible con educación primaria incompleta (51,1%), ocupación jubilada (28,6%), ingresos entre uno y dos salarios mínimos (48,9%), sin acceso a programas sociales (66,2%), vivienda propia (78,9%) y religión católica (79,7%), de acuerdo con la Tabla 1.

**Tabla 1:** Perfil sociodemográfico de las personas afectadas por la enfermedad de Hansen y en acompañamiento en la ESF (n* = 133). Campo Maior, PI, Brasil, 2023

Variables	n* (%)	IC-95%^†^	Media	Dp^‡^
Sexo				
Masculino	72 (54,1)	(45,7-62,4)		
Femenino	61 (45,9)	(37,6-54,3)		
Raza/color				
Blanca	23 (17,3)	(11,6-24,4)		
Parda	100 (75,2)	(66,6-81,3)		
Negra	10 (7,5)	(3,9-12,9)		
Rango de edad			49,2	17,8
≤ 19 años	10 (7,5)	(3,9-12,9)		
20-59 años	83 (62,4)	(54,0-70,3)		
≥60 años	40 (30,1)	(22,8-38,2)		
Estado civil				
Casado	81 (60,9)	(30,4-58,8)		
Soltero/Divorciado/Separado/Viudo	54 (39,1)	(21,4-50,3)		
Escolaridad				
Sin escolaridad	13 (9,8)	(5,6-15,7)		
Educación primaria incompleta	68 (51,1)	(42,7-59,5)		
Educación primaria completa	06 (4,5)	(1,9-9,1)		
Educación secundaria incompleta	12 (9,0)	(5,0-14,8)		
Educación secundaria completa	27 (20,3)	(14,1-27,7)		
Educación superior incompleta	01 (0,8)	(0,1-3,5)		
Educación superior completa	06 (4,5)	(1,9-9,1)		
Ocupación				
Estudiante	11 (8,3)	(4,5-13,9)		
Ama de casa	24 (18,0)	(12,2-25,2)		
Jubilado	38 (28,6)	(21,4-36,6)		
Desempleado	08 (6,0)	(2,9-11,0)		
Otros	52 (39,1)	(31,1-47,6)		
Ingreso mensual medio				
<1 SM^§^	40 (30,1)	(22,8-38,2)		
1-2 SM^§^	65 (48,9)	(40,5-57,3)		
≥2 SM^§^	11 (8,3)	(3,4-12,0)		
Sin ingresos	17 (12,8)	(7,9-19,2)		
Acceso a programas sociales				
Sí	45 (33,8)	(26,2-42,2)		
No	88 (66,2)	(57,8-73,8)		
Situación de la vivienda				
Casa propia	105 (78,9)	(71,4-85,2)		
Casa alquilada	15 (11,3)	(6,7-17,5)		
Casa cedida	11 (8,3)	(4,5-13,9)		
Otros	02 (1,5)	(0,3-4,7)		
Personas que viven en la casa			2,7	1,2
Religión				
Católica	106 (79,7)	(72,3-85,9)		
Evangélica	16 (12,0)	(7,3-18,4)		
Espírita	03 (2,3)	(0,6-5,9)		
Sin religión	05 (3,8)	(1,4-8,0)		
Otros	03 (2,3)	(0,6-5,9)		

*n = Tamaño de la muestra; ^†^IC-95% = Intervalo de confianza para la proporción; ^‡^DE = Desviación estándar; ^§^SM = Salario mínimo vigente = R$ 1.320,00, Brasil, 2023

La mayoría (98,5%) afirmó recibir orientación de profesionales sobre prácticas de autocuidado en la UBS, siendo el enfermero el profesional citado por el 99,2% de los entrevistados. 

La actitud de la población encuestada hacia el autocuidado fue clasificada como positiva en el 98,5% de los casos y negativa en el 1,5. En la clasificación de las prácticas de autocuidado, se investigaron tres segmentos: cara, manos y pies, y en ninguno de ellos se observó predominio de prácticas satisfactorias (Tabla 2).

**Tabla 2:** Clasificación de las prácticas de autocuidado de la muestra del estudio en relación con la cara, las manos y los pies (n* = 133). Campo Maior, PI, Brasil, 2023

Variables	n* (%)	IC-95%^†^	Media	Dp^‡^
Prácticas de autocuidado-cara			2,6	0,59
Insatisfactorias	66 (49,6)	(41,2-58,1)		
Necesita mejorar	61 (45,9)	(37,6-54,3)		
Satisfactorias	06 (4,5)	(1.9-9.1)		
Prácticas de autocuidado-manos			2,7	0,67
Insatisfactorias	57 (42,9)	(34,7-51,3)		
Necesita mejorar	59 (44,4)	(36,1-52,8)		
Satisfactorias	17 (12,8)	(7,9-19,2)		
Prácticas de autocuidado-pies			2,8	0,48
Insatisfactorias	39 (29,3)	(22,1-37,4)		
Necesita mejorar	87 (65,4)	(57,1-73,1)		
Satisfactorias	07 (5,3)	(2,4-10,1)		

*n = Tamaño de la muestra; ^†^IC-95% = Intervalo de confianza para la proporción; ^‡^DE = Desviación estándar

Entre los casos investigados, el 95,5% presentó la clasificación operacional de multibacilar, con baciloscopia negativa para el 70,7%, forma clínica dimorfa (90,2%) y GIF 1 al momento del diagnóstico en el 62,4%. La mayoría nunca presentó una reacción hansénica (80,5%), y entre los que la presentaron, el 18% fueron tipo 1, pero sin neuritis (88%), y con inicio del episodio reaccional observado al momento del diagnóstico. Las úlceras plantares y palmares estuvieron ausentes en el 88% de los investigados. Las enfermedades asociadas fueron Diabetes Mellitus (DM) (15,8%) e Hipertensión Arterial Sistémica (HAS) (30,8%). Otras enfermedades incluyeron hipertiroidismo, ansiedad, dislipidemia y cardiopatías (Tabla 3).

**Tabla 3:** Caracterización clínica de los casos de enfermedad de Hansen en el estudio (n* = 133). Campo Maior, PI, Brasil, 2023

Datos clínicos	n* (%)	IC-95%^†^
Clasificación operacional		
Paucibacilar	06 (4,5)	(1,9-9,1)
Multibacilar	127 (95,5)	(90,9-98,1)
Baciloscopia		
Positivo	12 (9,0)	(5,0-14,8)
Negativo	94 (70,7)	(62,6-77,9)
No realizada	27 (20,3)	(14,1-27,7)
Forma clínica		
Indeterminada	05 (3,8)	(1,4-8,0)
Tuberculoide	02 (1,5)	(0,3-4,7)
Dimorfa	120 (90,2)	(84,3-94,4)
Virchowiana	06 (4,5)	(1,9-9,1)
GIF^‡^ en el diagnóstico		
GIF^‡^ 0	39 (29,3)	(22,1-37,4)
GIF^‡^ 1	83 (62,4)	(54,0-70,3)
GIF^‡^ 2	11 (8,3)	(4,5-13,9)
Reacción hansénica		
Tipo I	24 (18,0)	(12,2-25,2)
Tipo II	01 (0,8)	(0,1-3,5)
Nunca presentó	107 (80,5)	(73,1-86,5)
Ya presentó en otro momento.	01 (0,8)	(0,1-3,5)
Inicio del episodio reaccionario		
Diagnóstico	13 (9,8)	(5,6-15,7)
Tratamiento	08 (6,0)	(2,9-11,0)
Post-alta	05 (3,8)	(1,4-8,0)
No aplica	107 (80,5)	(73,1-86,5)
Úlceras plantares/palmares		
Actual	07 (5,3)	(2,4-10,1)
Previo	01 (0,8)	(0,1-3,5)
Nunca presentó	117 (88,0)	(81,6-92,7)
No hay registro	08 (6,0)	(2,9-11,0)
Enfermedades asociadas		
Diabetes mellitus		
No	112 (84,2)	(77,3-89,6)
Sí	21 (15,8)	(10,4-22,7)
Hipertensión arterial		
No	92 (69,2)	(61,0-76,5)
Sí	41 (30,8)	(23,5-39,0)
Otros		
No	46 (34,6)	(26,9-42,9)
Sí	87 (65,4)	(57,1-73,1)
Neuritis		
Actual	13 (9,8)	(5,6-15,7)
Previo	02 (1,5)	(0,3-4,7)
No aplica	117 (88,0)	(81,6-92,7)
No hay registro	01 (0,8)	(0,1-3,5)

*n = Tamaño de la muestra; ^†^IC-95% = Intervalo de confianza para la proporción; ^‡^GIF = Grado de incapacidad física

En el modelo de regresión múltiple, sólo dos variables permanecieron estadísticamente asociadas a las prácticas de autocuidado insatisfactorias: tener úlceras plantares o palmares se asoció con una reducción del 10% en las probabilidades de presentar un autocuidado insatisfactorio (ORa = 0,90; p = 0,05), y la presencia de neuritis redujo las probabilidades en un 59% (ORa = 0,41; p = 0,04) (Tabla 4).

**Tabla 4:** Modelos logísticos de prácticas de autocuidado insatisfactorias en personas con la enfermedad de Hansen (n* = 133). Campo Maior, PI, Brasil, 2023

Variables	Análisis bivariado				Análisis múltiple			
	ORb*	IC 95%^†^		valor p^||^	ORa^¶^	IC 95%		valor p^||^
		LI^‡^	LS^§^			LI^‡^	LS^§^	
Datos sociodemográficos								
Sexo				0,23				
Masculino	1,57	0,74	3,33					
Femenino	1							
Raza/color								
Blanca	1,52	0,32	7,14	0,59				
Parda	1,71	0,45	6,53	0,43				
Negra	1							
Estado civil								
Casado/Unión estable	0,92	0,44	2,07	0,92				
Divorciado/Soltero/ Viudo	1							
Educación								
Sin escolaridad	1,66	0,19	14,05	0,63				
Educación primaria	1,04	0,17	6,09	0,96				
Educación secundaria	1,50	0,23	9,46	0,66				
Educación superior	1							
Ocupación								
Estudiante	0,98	0,22	4,23	0,98				
Ama de casa	0,89	0,30	2,61	0,89				
Jubilado	0,70	0,28	1,75	0,70				
Desempleado	1,10	0,19	6,13	1,10				
Otros	1							
Ingreso mensual medio								
<1 SM**	0,50	0,12	2,06	0,33				
1-2 SM**	0,44	0,11	1,73	0,24				
Sin ingresos	1							
Clasificación operacional								
Paucibacilar	-			0,99				
Multibacilar	1							
Forma clínica								
Indeterminada	2,00	0,12	31,97	0,62				
Tuberculoide	-			0,99				
Dimorfa	1,16	0,20	6,65	0,86				
Virchowiana	1							
GIF^††^ en el diagnóstico								
No	1							
Sí	1,01	0,48	2,48	0,81				
Reacción hansénica								
No	1				1			
Sí	0,36	0,14	0,88	0,02	-			0,99
Inicio del episodio reaccionario								
Diagnóstico	0,27	0,08	0,89	0,03	-			1,00
Tratamiento	0,32	0,07	1,375	0,12	-			1,00
Post-alta	0,48	0,07	3,04	0,43	-			1,00
No aplica	1				1			
Úlceras plantares/palmares								
No					1			1
Sí	0,38	0,09	1,64	0,09	0,90	0,16	4,80	0,05
Diabetes								
No	1							
Sí	1,04	0,37	2,92	0,93				
Hipertensión arterial								
No	1							
Sí	0,84	0,38	1,88	0,68				
Otros								
No	1							
Sí	1,27	0,58	2,75	0,54				
Neuritis								
No	1				1			
Sí	0,31	0,10	0,93	0,03	0,41	0,12	1,37	0,04
Actitud de autocuidado								
Actitud negativa	1,51	0,31	1,11	0,99				
Actitud positiva								

*ORb = Razón de probabilidades bruta; ^†^IC-95% = Intervalo de confianza para la proporción; ^‡^LI = Límite inferior; ^§^LS = Límite superior; ^||^valor p; ^¶^ORa = Razón de probabilidades ajustada; **SM = Salario mínimo vigente = R$ 1.320,00, Brasil, 2023; ^††^GIF = Grado de incapacidad física

## Discusión

En la población estudiada, el sexo masculino representó la mayoría de los casos nuevos de enfermedad de Hansen multibacilar, especialmente en el rango de edad de 20 a 59 años, con educación primaria incompleta y bajos ingresos familiares. La enfermedad de Hansen afecta a individuos susceptibles y en países endémicos, principalmente aquellos de América Latina, donde factores como el bajo nivel socioeconómico, la desnutrición, las poblaciones rurales dispersas y el acceso limitado e inadecuado a los servicios de salud para el diagnóstico y tratamiento, entre otras características, desempeñan un papel importante en la cadena epidemiológica de transmisión e incrementan la prevalencia oculta de la enfermedad. Además, las condiciones económicas pueden ser un obstáculo potencial para la adherencia y la finalización del tratamiento^17^ ^)-(^ ^22^.

La escolaridad también es un factor determinante para la efectividad de las acciones de educación para la salud, ya que actúa como mediadora en el proceso de comprensión de la enfermedad y en la adherencia al tratamiento. Se entiende que los individuos con un mayor nivel de educación tienden a tener mayor facilidad para dominar los conceptos relacionados con la prevención y la identificación temprana de signos y síntomas, lo que consecuentemente favorece las prácticas de autocuidado y reduce el riesgo de incapacidades físicas. Un estudio aleatorizado con 635 personas afectadas por la enfermedad de Hansen en Nigeria concluyó que la escolaridad, si bien puede estar relacionada con una mejor comprensión de la autoimagen y el afrontamiento, puede no proteger a las personas del estigma experimentado en la comunidad. Este hallazgo refuerza la necesidad de programas y campañas educativas mejoradas para abordar los conceptos erróneos y mejorar la concientización sobre la enfermedad de Hansen^23^.

Un hallazgo de este estudio que llamó la atención se refiere a la proporción de casos multibacilares con discapacidad física de grado 1, lo que refuerza la prevalencia oculta de la enfermedad asociada al diagnóstico tardío y/o a la preparación insuficiente de los profesionales de la APS para el diagnóstico correcto y oportuno de los casos^24^. Una encuesta poblacional realizada en China mostró que, a pesar del progreso en la reducción de la prevalencia de incapacidades físicas relacionadas con la enfermedad de Hansen, entre 1990 y 2020, la proporción de casos con incapacidades entre los afectados por la enfermedad sigue siendo alta, lo que indica una detección en etapas más avanzadas y una transmisión comunitaria aún persistente^25^. Es fundamental cualificar y ampliar las acciones de detección temprana, prevención y tratamiento oportuno, considerando que es la forma clínica con mayor importancia epidemiológica, pues elimina el mayor número de bacilos, lo que contribuye a mantener la cadena de transmisión^17^.

La baciloscopia fue realizada en la mayoría de los casos, obteniéndose resultados negativos. Tratándose de una prueba de laboratorio complementaria al diagnóstico clínico, cuyo objetivo es detectar la presencia de *M. leprae* en frotis de raspado intradérmico y estimar la carga bacilar del paciente. Un resultado positivo indica un caso multibacilar; un resultado negativo no descarta el diagnóstico de la enfermedad y su realización no es obligatoria para el diagnóstico clínico. Además, la prueba facilita el seguimiento del tratamiento, permitiendo comparar el índice bacilar al inicio y al final del mismo^1^ ^),(^ ^26^.

Es de destacar que las reacciones hansénicas estuvieron ausentes en la mayoría de los participantes, sin embargo, en aquellos que sí las presentaron, fueron de tipo I. La aparición de reacciones es una de las principales dificultades en el manejo clínico de las personas con enfermedad de Hansen, pues son episodios agudos e inflamatorios que pueden ocurrir antes, durante y después de la poliquimioterapia, y ocasionan incapacidades físicas irreversibles en los nervios cuando no se reconocen y tratan adecuadamente^27^.

La actitud de las personas afectadas por enfermedad de Hansen hacia el autocuidado fue mayoritariamente positiva. Por otro lado, predominaron las prácticas de autocuidado insatisfactorias. En general, se espera que una actitud favorable sea precursora de prácticas saludables. Sin embargo, no siempre existe coherencia entre lo que las personas creen y sus acciones prácticas, lo que puede entenderse como una disonancia entre actitud y comportamiento.

De acuerdo con la teoría de la Consistencia Cognitiva, una actitud es probablemente precursora de un patrón de comportamiento. Sin embargo, desde 1934, La Pierre ya se había referido a las inconsistencias entre actitudes y prácticas, señalando disparidades entre lo que se dice y lo que se hace. Reforzando esta idea, se afirma que la incoherencia cognitiva está presente en la mayoría de las personas, la mayor parte del tiempo, y en todas las personas, en algún momento. Por lo tanto, este enfoque fue cuestionado desde el punto de vista empírico y teórico^28^ ^)-(^ ^29^.

Por otra parte, el enfoque de Consistencia Contingente analiza la consistencia entre la actitud y la práctica del sujeto en relación al objeto y las circunstancias situacionales que lo rodean, señalando la importancia de la red de apoyo o soportes estructurales, considerando dos tipos de individuos: aquellos que mantienen una consistencia permanente entre actitudes y prácticas, independientemente de los soportes, y los ambivalentes, que cambian su comportamiento en función de soportes o presiones momentáneas^29^ ^)-(^ ^30^.

En resumen, según esta teoría de la consistencia contingente, para evitar disonancias entre actitudes y prácticas, es necesario considerar la situación en la que se encuentra el sujeto y solamente controlando los factores circunstanciales negativos, el sujeto puede alcanzar una actitud positiva que le proporcione mejores niveles de salud.

Desde esta perspectiva, es fundamental la ejecución del autocuidado como factor primordial en la prevención de incapacidades y en la responsabilidad de la persona por mantener su salud^9^ ^),(^ ^31^, sin embargo, existen contextos de vulnerabilidad social, donde barreras estructurales y falta de recursos dificultan la materialización de actitudes positivas en prácticas consistentes de autocuidado, las cuales pueden ser influenciadas por habilidades, limitaciones, condiciones culturales, sociales y económicas que interfieren directamente en la salud de quien la realiza^32^. En un estudio realizado en la provincia de Jiangsu, China, la capacidad de autocuidado de los grupos atendidos en servicios de salud y en el domicilio fue influenciada por las habilidades de autocuidado y la alfabetización en salud^33^.

De este modo, en salud, no es suficiente conocer solo las actitudes y prácticas del individuo en relación con una determinada situación, sino también las contingencias en las que se encuentra inmerso. Por lo tanto, aunque la persona afectada pueda estar a favor del autocuidado, puede que no comprenda exactamente cómo hacerlo ni cuál es la base de su implementación**.** Además, la ausencia de apoyo continuo y la falta de conocimientos técnicos sobre los cuidados necesarios refuerzan esta disociación entre actitud y práctica, evidenciando las lagunas en la educación en salud^34^.

Considerando que el estigma puede ser una barrera para la realización del autocuidado, la literatura evidencia que el apoyo entre pares y el apoyo psicosocial, por medio de grupos de autoayuda, contribuyen significativamente a su reducción entre las personas afectadas por la enfermedad de Hansen y la comunidad en la que se encuentran. Estos grupos pueden desempeñar un papel fundamental en las intervenciones para reducir el estigma sobre las enfermedades tropicales desatendidas, contribuyendo a un diagnóstico más rápido, una mayor adherencia al tratamiento y prácticas de autocuidado^23^ ^),(^ ^35^.

La enfermedad de Hansen sigue siendo un gran desafío para la salud pública nacional e internacional, considerando que es una enfermedad que afecta no solo la piel sino también los nervios periféricos, con una fuerte tendencia a generar deformidades e incapacidades, así como procesos inflamatorios (neuritis)^36^ ^)-(^ ^39^. Cabe destacar que las prácticas insatisfactorias se redujeron cuando los participantes presentaron úlceras plantares o palmares, o neuritis. Las demandas de autocuidado están directamente relacionadas con los ojos, la cara, las extremidades superiores e inferiores, especialmente las manos y los pies, considerando que estos son los principales sitios de afectación cutánea y neural^32^.

La neuropatía periférica es una de las principales manifestaciones de la enfermedad, ya que compromete las funciones sensoriales, motoras y autónomas, lo que resulta en deformidades visibles y deterioro de la función neural^1^ ^),(^ ^40^. Entre las deformidades físicas más comunes, las úlceras plantares son una de las principales. La región plantar es el sitio más afectado por las úlceras por el hecho de que las personas no perciben los traumatismos físicos como heridas y quemaduras, y las heridas permanecen continuamente expuestas al impacto de la locomoción y el contacto debido a la disminución o pérdida de la sensibilidad. El retraso en el diagnóstico es la principal razón del riesgo de desarrollar incapacidades, como las úlceras^41^ ^)-(^ ^42^.

El enfermero, como uno de los profesionales integrantes del equipo de APS, se convierte en un elemento clave en la ejecución de las acciones propuestas por el Programa Nacional para el Control y la Eliminación de la Enfermedad de Hansen (PNCEH), actuando en el cuidado integral de los pacientes, desde el diagnóstico hasta la finalización del tratamiento, además de guiar las prácticas de autocuidado^43^ ^)-(^ ^44^. Cabe destacar que la participación de profesionales capacitados con una comunicación efectiva facilita el reconocimiento temprano de signos y síntomas, el acceso a los servicios de salud, el estímulo del tratamiento y la motivación para la práctica del autocuidado con el fin de prevenir incapacidades relacionadas con la enfermedad de Hansen^45^ ^)-(^ ^46^.

En concordancia con lo anterior, una investigación realizada en los distritos de Dhanusa y Mahottari, Nepal para evaluar los conocimientos y las habilidades de 31 profesionales sanitarios locales concluyó que el desempeño medio general en la atención de la enfermedad de Hansen era bajo (53%) y podría mejorarse mediante la formación basada en la evidencia, seguimiento presencial, monitorización y supervisión, con el fin de facilitar la eliminación de la transmisión de la enfermedad de Hansen. Sin embargo, en el estudio se observó que el asesoramiento brindado por profesionales de la salud especializados mejoró significativamente las creencias y prácticas positivas de los pacientes con respecto al autocuidado durante el estudio^47^.

La enfermedad de Hansen, sin una intervención temprana, puede causar daños nerviosos irreversibles, deterioro e incapacidad, lo que implica no solo un problema de salud, sino también un problema social. Se evidencia el autocuidado como una de las principales estrategias en la prevención, el monitoreo, la gestión y el control de las discapacidades**,** siendo el individuo responsable de mantener y promover su propia vida, salud y bienestar^44^ ^),(^ ^48^.

El potencial incapacitante causado por la enfermedad es bastante significativo, y la evaluación de la incapacidad es importante para la educación y la promoción del autocuidado. La prevención se logra mediante procedimientos y ejercicios que la propia persona, debidamente guiada y capacitada, debe realizar regularmente en casa, durante el tratamiento y después del alta. Es fundamental un cambio de comportamiento, así como la inclusión del autocuidado en la rutina de las personas afectadas^49^.

El uso de Tecnologías Educativas contribuye a una mejora significativa en la adherencia al autocuidado de la cara, las manos y los pies. Además, mejora los índices de independencia en la realización de las actividades de la vida diaria, aumenta la frecuencia de las prácticas de autocuidado físico y mental, y la adherencia al tratamiento de la enfermedad de Hansen^50^.

Una de las limitaciones identificadas se refiere al abordaje de las personas que padecieron enfermedad de Hansen entre 2019 y 2023, es decir, en un período de hasta cinco años. Durante este período, es natural que se produjeran cambios de domicilio y fallecimientos. El tiempo también fue un factor que pudo haber contribuido a posibles sesgos de memoria. Además, la escasez de estudios sobre prácticas de autocuidado en la enfermedad de Hansen implicó algunas dificultades en las comparaciones; sin embargo, no perjudicó el estudio.

Este estudio hace valiosas contribuciones al campo del autocuidado en la enfermedad de Hansen, especialmente al reforzar la necesidad de intervenciones que vayan más allá del asesoramiento verbal, con foco en talleres demostrativos y en el seguimiento longitudinal de las habilidades de autocuidado que están siendo desarrolladas.

También contribuye al evidenciar que el enfoque de las acciones programáticas debe ser redirigido hacia el paciente sin incapacidades físicas o sin lesiones cutáneas aparentes, con el objetivo de romper el ciclo de agravamiento que puede llevar a daños irreversibles, promover el empoderamiento frente a la prevención de estas incapacidades por medio del autocuidado y posibilitar el acceso a los insumos necesarios para llevarlo a cabo de manera satisfactoria.

## Conclusión

Los resultados del estudio revelan que la mayoría de los participantes afectados por la la enfermedad de Hansen se encuentran en un contexto sociodemográfico y económico de desigualdad social, en el que prevalecen los bajos ingresos y el bajo nivel de educación. Si bien no se observó una asociación estadísticamente significativa entre los datos sociodemográficos y las prácticas de autocuidado de la cara, las manos y los pies, podría tener relevancia clínica. Se asume que la adopción y el desarrollo de estas actividades se ven influenciados por otros factores, como los relacionados con el equipo de atención médica y variables subjetivas.

La actitud frente a el autocuidado fue positiva en la gran mayoría de los participantes, sin embargo, predominaron las prácticas de autocuidado insatisfactorias, las cuales fueron significativamente reducidas en presencia de úlceras plantares o palmares, o neuritis.

Las acciones de educación en salud son estrategias primordiales para combatir la enfermedad de Hansen y deben ser implementadas por profesionales de atención primaria, quienes desempeñan un papel importante en la atención integral de los pacientes, tanto en la identificación temprana, la evaluación y la prevención de complicaciones, la adherencia al tratamiento y la promoción de la recuperación, así como el seguimiento de las necesidades de autocuidado. Por lo tanto, es necesario que estos profesionales tengan la capacitación adecuada.

La creación e implementación de grupos de ayuda mutua es una estrategia que brinda a las personas afectadas por la enfermedad de Hansen un espacio para compartir experiencias y conocimientos, desarrollar sus habilidades y ampliar su comprensión de los cuidados necesarios, enfocándose en la prevención de úlceras, secuelas e incapacidades.

## Data Availability

Todos los datos generados o analizados durante este estudio están incluidos en este artículo publicado.
